# Neuropeptide Ecdysis‐Triggering Hormone and Its Receptor Mediate the Fecundity Improvement of ‘*Candidatus* Liberibacter Asiaticus’‐Infected *Diaphorina citri* Females and *C*Las Proliferation

**DOI:** 10.1002/advs.202412384

**Published:** 2025-03-20

**Authors:** Xiaoge Nian, Bo Wang, Paul Holford, George Andrew Charles Beattie, Shijian Tan, Weiwei Yuan, Yijing Cen, Yurong He, Songdou Zhang

**Affiliations:** ^1^ Plant Protection Research Institute, Guangdong Academy of Agricultural Sciences, Key Laboratory of Green Prevention and Control on Fruits and Vegetables in South China Ministry of Agriculture and Rural Affairs Guangdong Provincial Key Laboratory of High Technology for Plant Protection Guangzhou 510640 P.R. China; ^2^ National Key Laboratory of Green Pesticide Department of Entomology College of Plant Protection South China Agricultural University Guangzhou 510642 China; ^3^ Department of Entomology and MOA Key Lab of Pest Monitoring and Green Management College of Plant Protection China Agricultural University Beijing 100193 China; ^4^ School of Science Western Sydney University Penrith NSW 2751 Australia

**Keywords:** candidatus liberibacter asiaticus, DcETH/DcETHR, diaphorina citri, endocrine network, mutualism

## Abstract

The severe Asiatic form of huanglongbing (HLB), caused by “*Candidatus* Liberibacter asiaticus” (*C*Las), threatens global citrus production via the citrus psyllid, *Diaphorina citri*. Culturing challenges of *C*Las necessitate reducing *D. citri* populations for disease management. *C*Las boosts the fecundity of *C*Las‐positive (*C*Las+) *D. citri* and fosters its own proliferation by modulating the insect host's juvenile hormone (JH), but the intricate endocrine regulatory mechanisms remain elusive. Here, it is reported that the *D. citri* ecdysis‐triggering hormone (DcETH) and its receptor *DcETHR* play pivotal roles in the reciprocal benefits between *C*Las and *D. citri* within the ovaries, influencing energy metabolism and reproductive development in host insects; miR‐210, negatively regulates *DcETHR* expression, contributing to this symbiotic interaction. *C*Las infection reduces 20‐hydroxyecdysone (20E) levels and stimulates DcETH release, elevating JH production via *DcETHR*, enhancing fecundity and *C*Las proliferation. Furthermore, circulating JH levels suppress 20E production in *C*Las*+* ovaries. Collectively, the orchestrated functional interplay involving 20E, ETH, and JH increases energy metabolism and promotes the fecundity of *C*Las*+ D. citri* and *C*Las proliferation. These insights not only broaden the knowledge of how plant pathogens manipulate the reproductive behavior of insect hosts but also offer novel targets and strategies for combatting HLB and *D. citri*.

## Graphical abstract

In *C*Las‐infected *D. citri*, *C*Las lowers 20E levels initially, relieving the suppression of ETH release. The subsequent increase in ETH levels upregulates its receptor *DcETHR* and activates the JH signaling pathway. The signaling cascade of 20E‐ETH/ETHR‐JH along with miR‐210, targeting *DcETHR*, boost female fecundity in *C*Las+ *D. citri* and promote *C*Las proliferation by affecting energy metabolism and *DcVgR* expression.

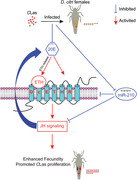

## 1 Introduction

Plant pathogenic microorganisms inhabiting phloem and xylem heavily rely on insect vectors for transmission in agricultural settings, with transmission often leading to substantial economic losses in crop production. Over the past few decades, the molecular interactions between plant pathogens and vector insects have emerged as a prominent research focus in fields such as microbiology, ecology, and entomology.^[^ ^1^, ^2^, ^3^ ^]^ However, to date, studies have predominantly concentrated on the win‐win relationship between plant viruses and pests with piercing‐sucking mouthparts; there has been little study of the molecular interplay between plant pathogenic bacteria and vector insects.^[^ ^4^, ^5^, ^6^ ^]^ Huanglongbing (HLB) currently stands as the most severe threat to the global citrus industry.^[^ ^7^ ^]^ The most severe form of HLB is attributed to the phloem‐colonizing bacterium, “*Candidatus* Liberibacter asiaticus” (*C*Las) that is primarily transmitted by the Asiatic citrus psyllid (*Diaphorina citri* Kuwayama (Hemiptera: Sternorrhyncha: Psyllidae) in a persistent manner.^[^ ^8^, ^9^ ^]^ Previous investigations highlighted a notable increase in the fecundity of *C*Las‐positive (*C*Las*+*) *D. citri* females compared to their *C*Las‐negative (*C*Las‐) counterparts resulting in greater population growth rates.^[^ ^10^, ^11^, ^12^ ^]^ Subsequent studies have shown how *C*Las manipulates juvenile hormone (JH) signaling in host insects to enhance the fecundity of *D. citri* females, thereby facilitating their own proliferation.^[^ ^13^ ^]^ Nevertheless, further in‐depth research is warranted to elucidate the intricate mechanisms through which the endocrine signaling pathways of host insects respond to *C*Las, fostering mutually beneficial outcomes.

Plant pathogenic microorganisms modify the physiology and development of host insects by influencing key biological traits, such as reproductive and feeding behaviors, thereby fostering a mutualistic relationship between them.^[^ ^1^ ^]^ The reproductive success of female insects is predominantly regulated by intricate endocrine signaling networks involving JH, ecdysis triggering hormone (ETH), and 20‐hydroxy ecdysone (20E).^[^ ^14^ ^]^ JH, a sesquiterpenoid hormone, is synthesized and secreted by the corpora allata (CA) of insects and is recognized as the primary hormone governing female insect reproduction.^[^ ^15^, ^16^ ^]^ The synthesis and release of JH by the CA is modulated by various neuronal and hormonal factors.^[^ ^17^ ^]^ ETH, an amidated linear peptide consisting of 11–34 amino acids, was initially identified in the hemolymph of the tobacco hornworm, *Manduca sexta* (L.) (Lepidoptera: Sphingidae), where it undergoes significant fluctuations during metamorphosis and acts as an ecdysis trigger.^[^ ^18^ ^]^ In *Drosophila melanogaster* (Meigen) (Diptera: Drosophilidae), ETH persists into adulthood, serving as a necessary allatotropin to stimulate JH production and reproduction, and a substantial regulatory impact of 20E on both ETH and the ETH receptor (ETHR) has been observed in adult insects.^[^ ^19^ ^]^ Maintaining a balance between 20E and JH is crucial for controlling oogenesis, with ETH playing a pivotal role in preserving this equilibrium to ensure successful oogenesis in female insects.^[^ ^19^ ^]^ Therefore, investigating whether the enhanced fecundity of *C*Las+ *D. citri* females is linked to coordinated functional networks involving 20E, ETH, and JH is of significant scientific relevance.

Insect endocrine hormones play a pivotal role in mediating the interactions between host insects and pathogens. For example, in *Helicoverpa armigera* (Hübner) (Lepidoptera: Noctuidae), the *H. armigera* single nucleopolyhedrovirus (HaSNPV) manipulates the crosstalk between 20E and JH pathways to regulate the tree‐top disease of HaSNPV‐infected larvae, facilitating virus proliferation and transmission.^[^ ^20^ ^]^ Also, to enhance infection, the entomopathogenic fungus *Metarhizium rileyi* (Farl.) Kepler, Rehner & Humber (Hypocreales: Clavipitaceae) deactivates ecdysone (20E) and suppresses the antimicrobial immune responses of *Galleria mellonella* (L.) (Lepidoptera: Pyralidae).^[^ ^21^ ^]^ In *C*Las+ *D. citri*, infection with *C*Las increases the JH signal by modulating the JH‐Met‐Kr‐h1 pathway thereby decreasing miR‐275 expression, leading to increased expression of *DcVgR* (the gene encoding the vitellogenin receptor) and enhanced female fecundity.^[^ ^13^ ^]^ Despite being recognized as a significant neuroendocrine peptide, the function of ETH in *D. citri* remains unexplored, with only its nucleotide sequences being documented.^[^ ^22^ ^]^ Building upon the elucidation of the JH signaling pathway, investigating the role of ETH in boosting the fecundity of *C*Las+ *D. citri* females represents a novel and innovative research avenue. Furthermore, microRNAs (miRNAs), a class of endogenous, 18–25 nucleotide, non‐coding RNAs, play a critical role in inhibiting mRNA translation or promoting degradation at the transcriptional or post‐transcriptional level.^[^ ^23^ ^]^ miRNAs also play essential roles in insect reproduction and in the intricate interplay between host insects and pathogens.^[^ ^24^, ^25^ ^]^ For instance, miR‐8 and miR‐429 target the BrZ2 gene implicated in HaSNPV‐induced tree‐top disease in *H. armigera* larvae.^[^ ^20^ ^]^ Similarly, miR‐275 targets *DcVgR* suppressing its expression thereby increasing fecundity the of *C*Las+ *D. citri* females and enhancing multiplication of *C*Las.^[^ ^13^ ^]^ Therefore, we postulate that miRNAs may target other genes involved in endocrine hormone signaling to participate in the win‐win strategy between *C*Las and *D. citri* within the ovaries.

Here, we aimed to elucidate the role of the host endocrine network in the enhanced fecundity of *C*Las+ *D. citri* females and *C*Las proliferation. Initially, we employed fluorescence in situ hybridization (FISH), calcium imaging, and RNA interference (RNAi) techniques to investigate the impact of *DcETH* and its receptor, *DcETHR*, on energy metabolism, reproductive parameters, and *C*Las levels. Subsequently, through a combination of in vivo and in vitro assays, we demonstrated that miR‐210 targets *DcETHR*, contributing to the increased fecundity of *C*Las+ *D. citri* females and *C*Las proliferation. Finally, we examined the coordinated functional networks involving 20E, ETH, and JH in the context of increased fecundity of *C*Las+ *D. citri* females and *C*Las proliferation.

## 2 Results

### 2.1 *C*Las Infection Increases the Levels of *DcETH* and Its Receptor *DcETHR* in *D. citri*

**Figure 1:**
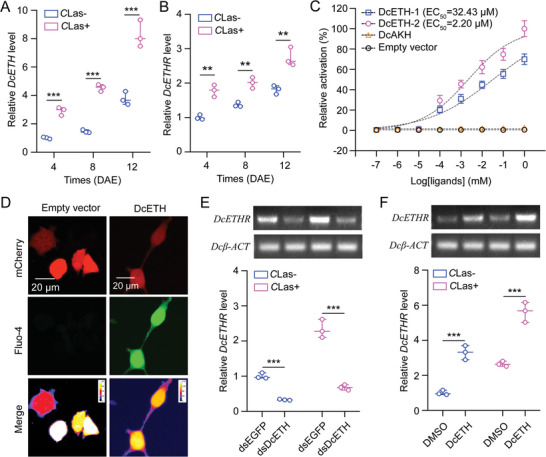
*C*Las infection increases the levels of *DcETH* and its receptor *DcETHR* in *D. citri*. A‐B) Effect of *C*Las infection on the mRNA expression of *DcETH* and *DcETHR* at various development stages of *C*Las+ females compared to *C*Las‐ individuals. *C*Las+ and *C*Las‐ denote *C*Las‐positive and *C*Las‐negative *D. citri* females, separately. DAE represents days after eclosion. C) Fluo‐4 AM fluorescence signals were observed upon the introduction of *DcETHR* into mammalian HEK293T cells that were subsequently treated with mature peptides of DcETH. A recombinant plasmid containing the complete ORF sequence of *DcETHR* was generated by inserting it into the pcDNA3.1(+)‐mCherry plasmid. Negative controls included the use of an empty vector or DcAKH treatment. D) Representative images showing Ca^2+^ activity subsequent to the introduction of *DcETHR* into mammalian HEK293T cells and exposure to mature peptide treatments of DcETH. In the control group labeled “Empty vector”, cells were transfected with the empty pcDNA3.1(+)‐mCherry vector. The scale bar represents 20 µm. E‐F) Influence of *DcETH* knockdown and DcETH mature peptide treatments on the mRNA expression of *DcETHR* in both *C*Las+ and *C*Las‐ females. Results in A‐B and E‐F were represented as means ± SEs with three independent biological replicates. Statistically significance was determined using pair‐wise Student's *t*‐test, with levels of significance denoted by ** (*p* < 0.01) and *** (*p* < 0.001).

To explore the function of ETH signaling in the interaction between *D. citri* and *C*Las, we retrieved the sequence of *D. citri* ETH, designated as *DcETH* (MG550169.1), from the GenBank database.^[^ ^22^ ^]^ In our quest to identify the receptor for *DcETH*, we discovered a sequence (MG550195.1) in *D. citri* with similarity to other “ETHRs” by mining the GenBank database, which we named *DcETHR*. Sequence analysis indicated that the open reading frame (ORF) of *DcETH* comprises 459 base pairs (bp), encoding a predicted polypeptide of 152 amino acids (aa) with a 20 aa signal peptide and two mature peptides. The ORF of *DcETHR* spans 3558 bp, encoding 1185 aa with seven transmembrane domains and a molecular weight of 134.32 kDa. Comparative analysis showed limited similarities between the amino acid sequence of DcETH and “ETHs” from five other selected insect species, except for the second mature peptide, which showed substantial similarity with the other species (Figure S1A, Supporting Information). An unrooted phylogenetic tree shows that the DcETH protein is most closely related to analogs from five selected hemipteran species (ApETH, SgETH, PsETH, NlETH, and SfETH) but differs from analogs in Lepidoptera (HaETH and BmETH), Coleoptera (TcETH), and Diptera (CqETH and BdETH) (Figure S1B, Supporting Information). Comparative analysis revealed a high amino acid identity among the seven transmembrane domains of DcETHR and its homologues in five other insect species (Figure S2A, Supporting Information). Phylogenetic analysis (Figure S2B, Supporting Information) indicated a close evolutionary relationship between the DcETHR protein sequence and homologues of SgETHR and TcETHR. The tertiary structure of DcETHR was modeled, and its interaction with DcETH was simulated utilizing the Phyre^2^ online server and refined using PyMOL‐v1.3r1 software (Figure S2C, Supporting Information). Spatial expression analysis indicated higher expression of both *DcETH* and *DcETHR* in the head compared to that in the midgut, ovary, and fat bodies (Figures S1C and S2D, Supporting Information). qRT‐PCR results demonstrated an increase in *DcETH* or *DcETHR* expression following female adult eclosion, with *C*Las infection markedly elevating its expression 4, 8, and 12 days after eclosion (DAE) in *C*Las+ females compared to *C*Las‐ individuals (**Figure** **1**A,B).

Given that ligand binding to G‐protein coupled receptors triggers calcium ion influx, we conducted cell‐based calcium mobilization assays and calcium imaging to further validate the activation of *DcETHR* by DcETH. The results demonstrated a dose‐dependent activation of *DcETHR* by the two mature peptides of DcETH, with EC_50_ values of 32.43 µM for DcETH‐2 and 2.20 µM for DcETH‐1, respectively (Figure 1C). Conversely, minimal calcium influx was observed in DcETHR‐expressing cells treated with the DcAKH mature peptide or an empty vector. Calcium imaging data revealed a significant increase in the intracellular Ca^2+^ signal in DcETHR‐expressing cells upon treatment with mature peptide of DcETH‐2, contrasting with the absence of any signal in empty vector‐treated group (Figure 1D). Furthermore, RNAi‐mediated *DcETH* knockdown led to a notable decrease in *DcETHR* expression compared to dsEGFP treatment, while treatment with DcETH‐2 resulted in a clear increase in *DcETHR* expression compared to DMSO treatment in both *C*Las‐ and *C*Las+ females (Figure 1E,F). Overall, our in vitro and in vivo findings strongly support the role of *DcETHR* as the receptor for *DcETH*.

### 2.2 *DcETH* and Its receptor *DcETHR* are Essential for the Enhanced Fecundity of *D. citri* Females Infected by *C*Las and *C*Las Proliferation

**Figure 2:**
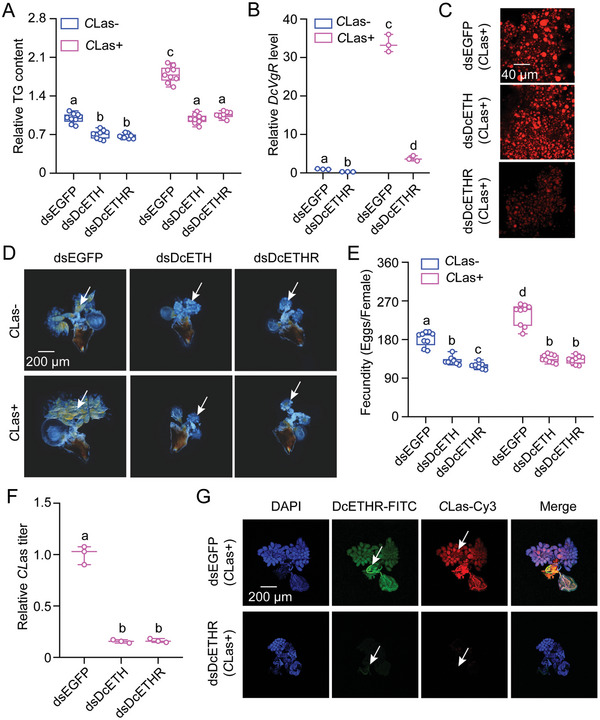
*DcETH* and its receptor *DcETHR* are essential for the enhanced fecundity of *D. citri* females infected by *C*Las and *C*Las proliferation. A) The impact of *DcETH* and *DcETHR* knockdown on TG contents in *C*Las+ or *C*Las‐ females. TG stands for triglyceride. B) Relative mRNA levels of *DcVgR* after *DcETHR* knockdown at 48 h in both *C*Las+ and *C*Las‐ females. C‐G) Effect of *DcETH* and *DcETHR* knockdown on lipid droplets size, ovary development, egg laying numbers, and *C*Las levels in *C*Las+ or *C*Las‐ females. The scale bar for lipid droplets is 40 µm and for the ovary is 200 µm. DAPI staining was used to visualize cell nuclei in blue. *DcETHR*‐FITC shown that the *DcETHR* signal visualized in green by labeling with FITC. *C*Las‐Cy3 labeling was used to detect the *C*Las signal and visualized in red. The merged image combined the imaging of DAPI, *DcETHR*‐FITC, and *C*Las‐Cy3 signals. The data in B and F are mean ±SEs with three independent biological replicates. Results in A and E were displayed as mean ± SE with nine independent biological replicates. The data were subjected to ANOVA followed by a Tukey's Honest Significant Difference tests. Different letters above the bars denote statistically significant differences at *p* < 0.05.

To elucidate the impact of *DcETH* and *DcETHR* on the enhanced fecundity of *C*Las+ females and *C*Las proliferation, RNA interference (RNAi) assays were performed as per.^[^ ^13^ ^]^ After dsRNA administration for 48 h, the transcript levels of *DcETH* in the ovaries of both *C*Las‐ and *C*Las+ individuals markedly deceased by 70.17% and 73.57%, while knockdown of *DcETHR* resulted in a 48.53% and 82.72% decrease in the mRNA levels of *DcETHR*, respectively, in comparison to females fed with dsEGFP (Figure S3A,B Supporting Information). Moreover, the reduction in *DcETH* or *DcETHR* expression via RNAi led to reduced triglyceride (TG) contents, decreased expression of *DcVgR*, *DcVg1*, and *DcVg2*, and smaller lipid droplets in both *C*Las‐ or *C*Las+ females compared to those treated with dsEGFP (**Figure** **2**A–C; Figure S4A,B, Supporting Information). Notably, successful interference with *DcETH* or *DcETHR* expression impeded ovarian development (Figure 2D), reduced egg laying (*DcETH*: from 184.94 to 131.17 and 120.38 in *C*Las‐ females; from 236.29 to 136.61 and 132.67 in *C*Las+ psyllids) (Figure 2E), and reduced the relative *C*Las titer and signals in *C*Las+ ovaries compared to dsEGFP treatments (Figure 2F,G; Figure S5, Supporting Information). These findings indicate that host *DcETH* and *DcETHR* play important roles in response to *C*Las infection, enhancing *D. citri* fecundity and promoting *C*Las proliferation.

### 2.3 Identification and Validation of miR‐210 Directly Targets *DcETHR*

**Figure 3:**
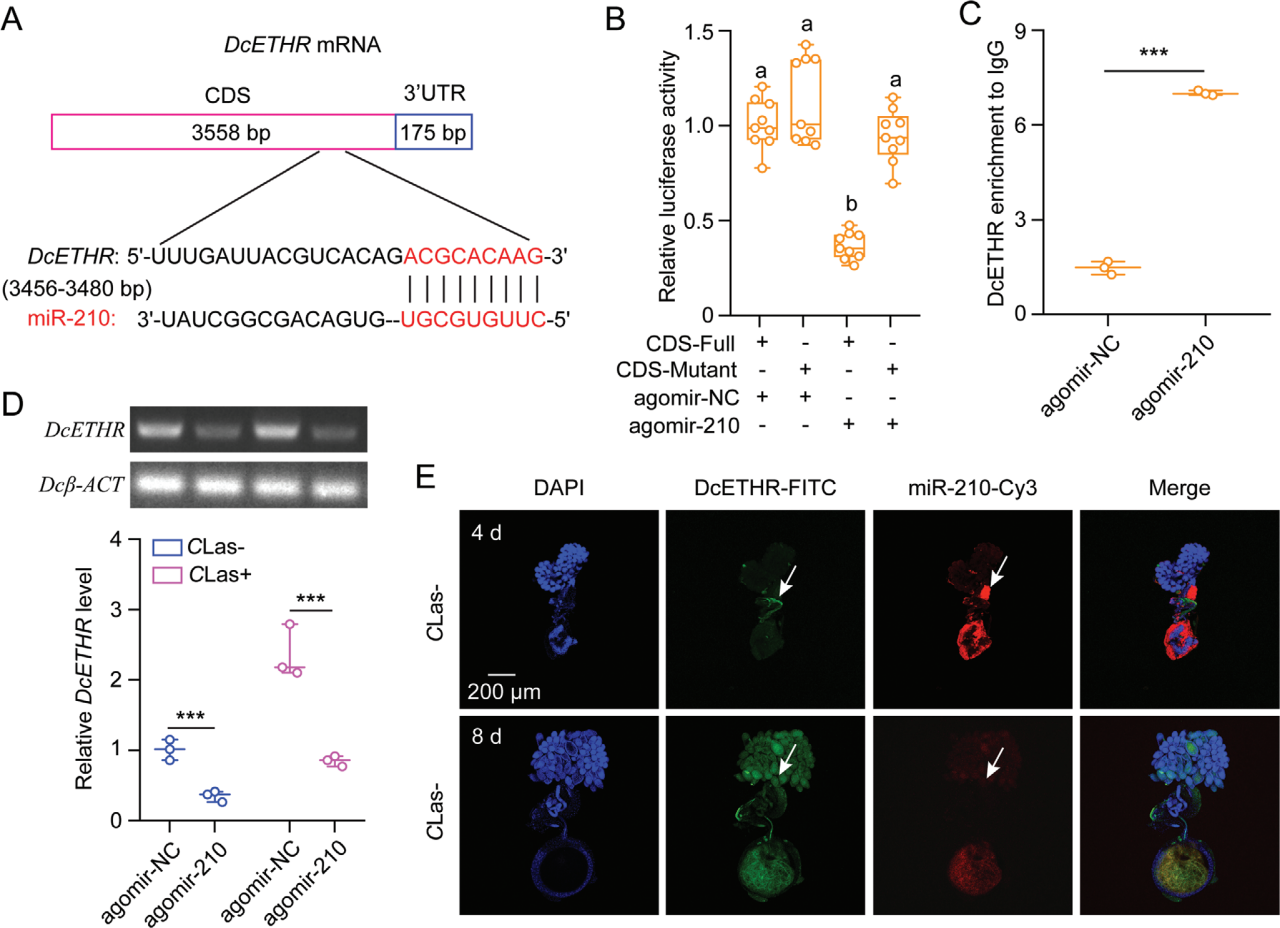
Confirming of miR‐210 directly targets *DcETHR*. A) Putative binding sites of miR‐210 in the mRNA sequence of *DcETHR* predicted using miRanda and Targetscan algorithms. B) Validation of the target interactions between miR‐210 and *DcETHR* was carried out through dual luciferase reporter assays in vitro. C) In vivo validation of miR‐210 targeting *DcETHR* was conducted using RNA‐binding protein immunoprecipitation (RIP) assays. D) The influences of agomir‐210 treatments on *DcETHR* mRNA expression by semi‐quantitative RT‐PCR and qRT‐PCR. E) Representative confocal images of miR‐210 and *DcETHR* in *C*Las‐nagetive ovaries at 4 and 8 DAE. Scale bar is 200 µm. DAPI staining in blue was used for visualizing cell nuclei, *DcETHR*‐FITC shown that the *DcETHR* signal visualized in green by labeling with FITC, while miR‐210‐Cy3 labeling highlighted the miR‐210 signal in red. The merged image combined DAPI, *DcETHR*‐FITC, and miR‐210‐Cy3 signals for a comprehensive analysis. Data in B were indicated as mean ± SE with nine independent biological replicates, and subjected to ANOVA followed by a Tukey's Honest Significant Difference tests. Different letters above the bars denote statistically significant differences at *p* < 0.05. Results in C and D were displayed as means ± SEs with three independent biological replicates, while statistical significance was evaluated using pair‐wise Student's *t*‐test, with significance levels represented by *** (*p* < 0.001).

To investigate the post‐transcriptional regulation of the *DcETHR* gene, we utilized two software tools, miRanda and Targetscan, to predict possible miRNAs that could interact with this gene; these programs identified four miRNAs (miR‐210, miR‐317, miR‐31b, and miR‐2b) with the potential to bind to sites in the coding regions of *DcETHR* (**Figure** **3**A; Figure S6A, Supporting Information). Interestingly, no miRNAs were identified in the 3′ untranslated region (UTR) as the 3′UTR sequence was short. Results from in vitro dual‐luciferase reporter assays revealed a significant decrease in relative luciferase activities upon co‐transfection of agomir‐210 and DcETHR‐CDS‐pmirGLO, while no change was observed with the other three miRNAs (Figure 3B; Figure S6B, Supporting Information). Notably, luciferase activity returned to control levels when using a mutated CDS sequence lacking the binding sites of miR‐210. Furthermore, RNA immunoprecipitation assay data demonstrated a significant increase in the transcripts of *DcETHR* and miR‐210 by ≈4.74‐fold and 2.87‐fold, respectively, in nymphs fed with agomir‐210 when compared to an IgG control (Figure 3C; Figure S7A, Supporting Information). Semi‐quantitative RT‐PCR and qRT‐PCR indicated that agomir‐210 treatments inhibited *DcETHR* expression compared to the agomir‐NC control in *C*Las‐ or *C*Las+ females (Figure 3D). Additionally, FISH results showed that miR‐210 exhibited contrasting expression patterns with *DcETHR* in *C*Las‐ females at 4 and 8 DAE (Figure 3E). Collectively, these data confirm that miR‐210 directly targets *DcETHR*.

### 2.4 MiR‐210 Directly Targets *DcETHR* to Modulate the Mutualistic Interactions between *D. citri* and *C*Las within the Ovaries

**Figure 4:**
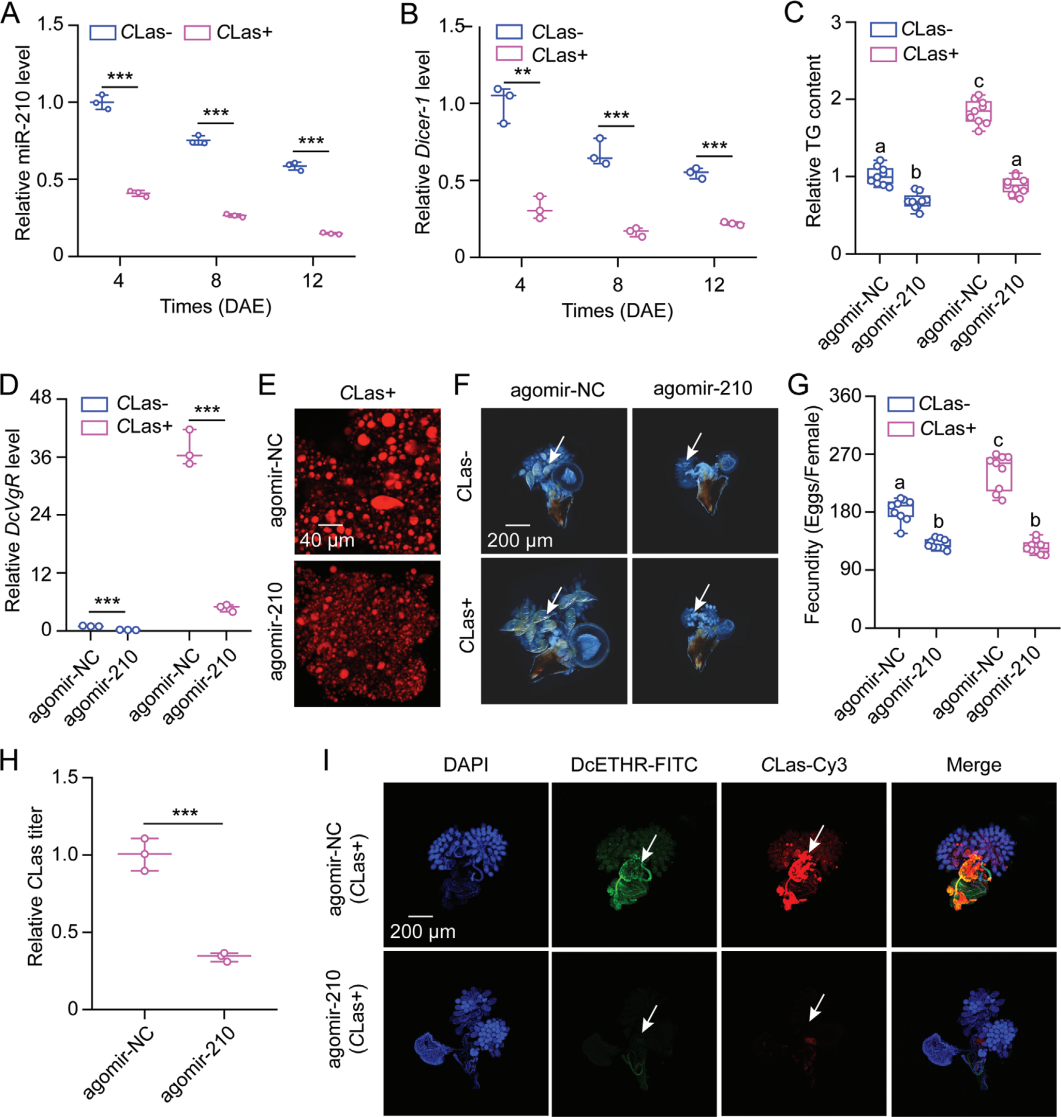
miR‐210 modulates the mutualistic interactions between *D. citri* and *C*Las within the ovaries. A‐B) Influence of *C*Las infection on the mRNA expression of miR‐210 and *Dicer‐1* at 4, 8, and 12 DAE of *C*Las+ females compared to *C*Las‐ individuals. C‐I) Effect of agomir‐210 treatments on TG content, *DcVgR* mRNA levels, lipid droplets size, ovary development, egg laying numbers, *C*Las titer, and *C*Las proliferation in *C*Las+ or *C*Las‐ females compared to agomir‐NC treatments. The scale bar for lipid droplets was set at 40 µm, and for the ovary at 200 µm. DAPI staining in blue, *DcETHR*‐FITC shown in green, and *C*Las‐Cy3 labeling in red are same as described in Figure 2. Data in A, B, D, and H were indicated as mean ± SEs with three independent biological replicates, and evaluated using pair‐wise Student's *t*‐test, with significance levels represented by ** (*p* < 0.01) and *** (*p* < 0.001). Results in C and G were displayed as means ± SEs with three independent biological replicates, and subjected to ANOVA followed by a Tukey's Honest Significant Difference tests. Different letters above the bars denote statistically significant differences at *p* < 0.05.

The qRT‐PCR findings revealed a decrease in miR‐210 expression at 4, 8, and 12 DAE in *C*las‐ females compared to *C*Las+ individuals (**Figure** **4**A), which was inversely correlated with *DcETHR* expression. miR‐210 exhibited ubiquitous expression across the four examined tissues, with the highest levels detected in the head (Figure S7B, Supporting Information). To understand how *C*Las infection impacts miR‐210 expression, we also assessed the transcript levels of two genes involved in miRNA synthesis and function. The mRNA levels of *Dicer‐1* were shown a decrease in *C*Las+ females compared to *C*Las‐ individuals (Figure 4B), whereas the expression of *Ago‐1* remained unchanged following *C*Las infection in *D. citri* females (Figure S7C, Supporting Information). These results suggest that *C*Las infection may reduce miR‐210 expression by affecting the expression of the Dicer enzyme during miRNA synthesis.

Consistent with expectations, agomir‐210 treatment markedly resulted in decreased TG content, reduced *DcVgR* expression, smaller lipid droplets size, delayed ovarian development, reduced egg production, lower *C*Las levels, and *DcETHR* signal in both *C*Las‐ or *C*Las+ females compared to the agomir‐NC treatments (Figure 4C–I; Figure S7D, Supporting Information). Overall, our results suggest that miR‐210 plays a crucial role in enhancing the female fecundity of *D. citri* in response to *C*Las infection and facilitating *C*Las proliferation by targeting *DcETHR* gene.

### 2.5 20E Regulates ETH Signaling in Fecundity Enhancement of *D. citri* and *C*Las Proliferation

**Figure 5:**
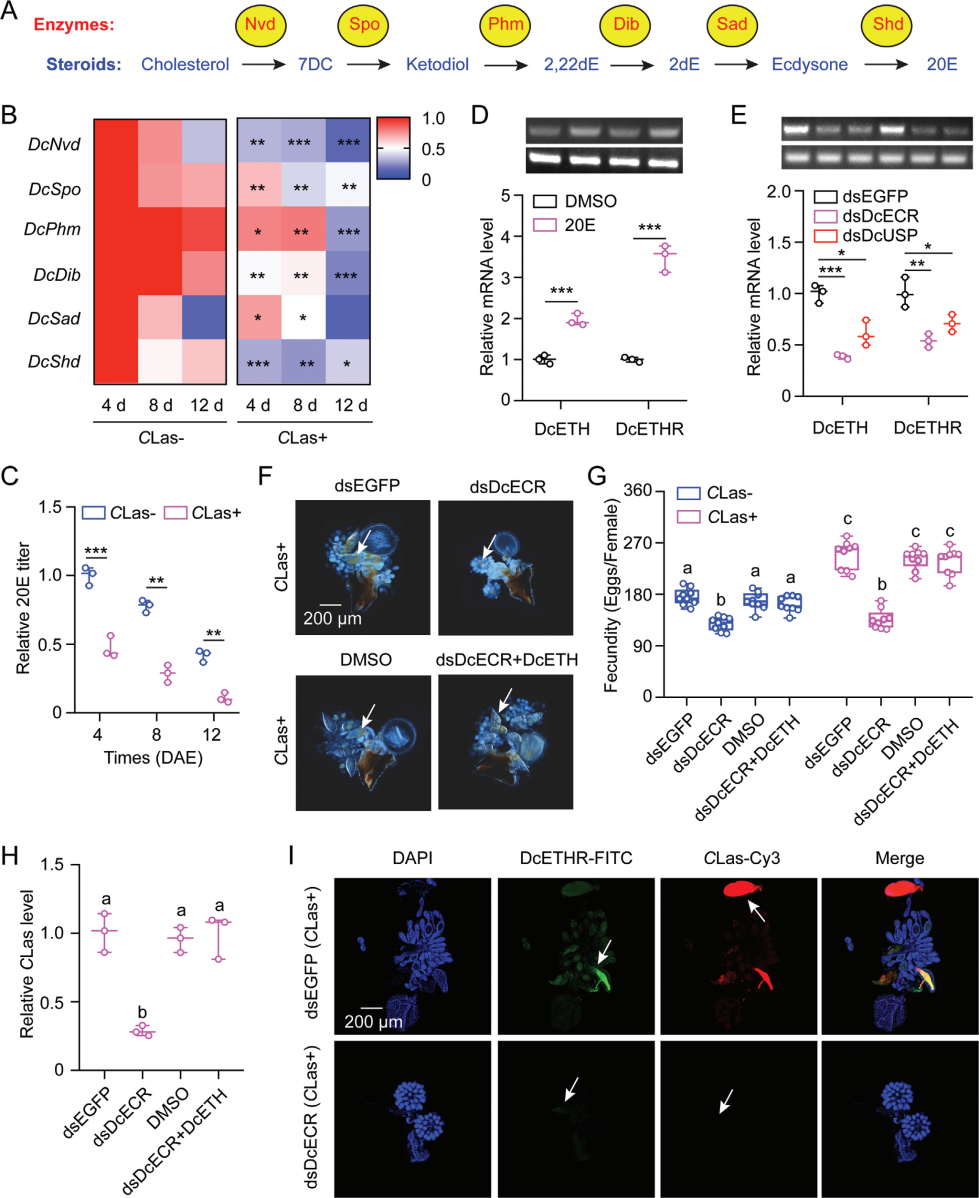
ETH signaling acts downstream of 20E signaling to mediate the mutualistic interactions between *D. citri* and *C*Las within the ovaries. A) Diagram of genes associated with 20E biosynthesis pathway in insects. Nvd: Neverland; Spo: Spook; Phm: Phantom; Dib: Disembodied; Sad: Shadow; Shd: Shade. B) The heat map of expression patterns of 20E biosynthetic genes at 4, 8, and 12 DAE of *C*Las+ females compared to *C*Las‐ individuals. C) The impact of *C*Las infection on the relative 20E titer at various developmental stages of *C*Las+ females compared to the *C*Las‐ individuals. D‐E) Analysis of *DcETH* and *DcETHR* expression following 20E treatments and deficiency of 20E receptors in both *C*Las+ females. F‐I) The influences of *DcECR* knockdown on ovary development, egg laying numbers, *C*Las titer, and *C*Las proliferation in *C*Las+ or *C*Las‐ females. “dsDcECR+DcETH” denotes treatment with DcETH to rescue the *DcECR* knockdown. Data in panels A and C‐E were presented as means ± SEs based on three independent biological replicates, and evaluated using pair‐wise Student's *t*‐test, with significance levels denoted by * (*p* < 0.05), ** (*p* < 0.01), and *** (*p* < 0.001). Results in panels G were displayed as means ± SEs from nine independent biological replicates, and subjected to ANOVA followed by a Tukey's Honest Significant Difference tests. Different letters above the bars denote statistically significant differences at *p* < 0.05.

During juvenile stages, ecdyteroids such as 20E stimulate the synthesis of ETH in Inka cells but inhibit its release.^[^ ^19^, ^26^ ^]^ Therefore, we sought to investigate whether the ETH/ETHR signaling pathway is modulated by 20E in *C*Las+ females. To evaluate the impact of *C*Las infection on 20E biosynthesis, we examined the expression profiles of six genes: *Neverland* (*Nvd*), *Spook* (*Spo*), *Phantom* (*Phm*), *Disembodied* (*Dib*), *Shadow* (*Sad*) and *Shade* (*Shd*) (**Figure** **5**A).^[^ ^27^ ^]^ The qRT‐PCR results depicted in a heatmap indicated a progressive downregulation of all six genes at 4, 8, 12 DAE in *C*Las+ females in comparison to *C*Las‐ individuals (Figure 5B). The 20E titer was also lower in *C*Las+ females compared to these *C*Las‐ counterparts, and in both groups of insects, they declined over time following eclosion (Figure 5C). mRNA levels of its receptors (*DcECR* and *DcUSP*) in *C*Las+ ovaries were obviously lower in *C*Las+ psyllids compared to *C*Las‐ individuals (Figure S8A,B, Supporting Information). qRT‐PCR results demonstrated that 20E treatments markedly increased the expression of *DcETH* and *DcETHR* (Figure 5D); successful knockdown of *DcECR* or *DcUSP* in *C*Las‐ and *C*Las+ females (Figure S8C,D, Supporting Information) led to inhibition of both *DcETH* and *DcETHR* expression compared to dsEGFP treatments (Figure 5E), suggesting a role for 20E signaling in promoting the release of ETH and regulating the gene expression of *DcETH* and *DcETHR*.

Furthermore, RNAi‐mediated knockdown of *DcECR* significantly impaired ovarian development in *C*Las+ females, reduced egg laying in both *C*Las‐ and *C*Las+ females, decreased *C*Las levels, and weaker signals of *DcETHR* in *C*Las+ ovaries, while treatment with DcETH rescued the phenotypic defects induced by *DcECR* knockdown (Figure 5F–I). Collectively, these findings indicate that 20E plays a regulatory role in ETH signaling causing the increase in fecundity enhancement of *C*Las+ psyllids and *C*Las proliferation.

### 2.6 ETH Signaling is an Upstream Regulator of JH Signaling

**Figure 6:**
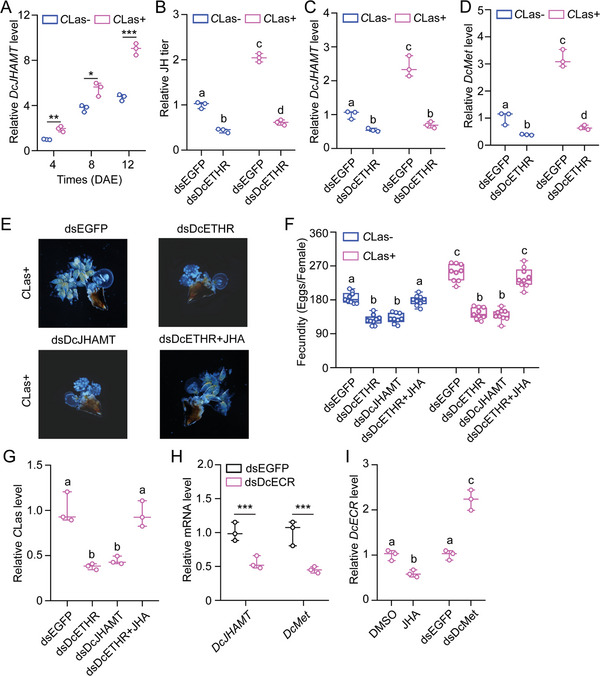
ETH signaling serves as the upstream regulator of JH signaling in the regulation of mutualistic interactions between *D. citri* and *C*Las within the ovaries. A) The effect of *C*Las infection on the relative mRNA expression of *DcJHAMT* at different developmental stages of *C*Las+ females compared to *C*Las‐ individuals. B‐D) The influences of RNAi‐mediated *DcETHR* knockdown on relative JH titer, mRNA expression of *DcJHAMT* and *DcMet* in both *C*Las+ and *C*Las‐ females. E‐G) JHA treatments rescued the impaired ovary development, egg laying numbers, and *C*Las titer caused by *DcETHR* knockdown in *C*Las+ females. H) RNAi‐mediated knockdown of *DcECR* reduced the mRNA expression of *DcJHAMT* and *DcMet* in *C*Las+ females. I) Effect of JHA treatment and *DcMet* knockdown on the transcription of *DcECR* in *C*Las+ psyllids. Data in A‐D and G‐I were presented as mean ± SE with three independent biological replicates. Results in F were shown as mean ± SE with nine independent biological replicates. The statistical significance of the data in panels A and H was assessed using pair‐wise Student's *t*‐test, with significance levels indicated by * (*p* < 0.05), ** (*p* < 0.01), and *** (*p* < 0.001). The data in panels B‐D, F, G, and I were subjected to ANOVA followed by a Tukey's Honest Significant Difference tests. Different letters above the bars denote statistically significant differences at *p* < 0.05.

In this study, we aimed to determine the interplay between JH signaling and *DcETH*/*DcETHR* in *C*Las+ ovaries. qRT‐PCR revealed a significant higher expression of the JH biosynthesis gene, *DcJHAMT*, in *C*Las+ females compared to *C*Las‐ females (**Figure** **6**A). Expression of *DcJHAMT* increased with time after eclosion, particularly in the *C*Las+ psyllids. Knockdown of *DcETHR* led to a marked decrease in JH titer, *DcJHAMT* expression, and *DcMet* expression in both *C*Las‐ and *C*Las+ females (Figure 6B–D), indicating that *DcETH*/*DcETHR* acts as the upstream regulator of JH signaling. Additionally, knockdown of *DcJHAMT* resulted in delayed ovarian development, decreased egg laying, and reduced *C*Las titers in *C*Las+ females, similar to the effects observed with *DcETHR* interference. Treatment with JH analogue (methoprene, JHA) rescued the phenotypic defects caused by *DcETHR* knockdown (Figure 6E–G), further supporting the role of *DcETH*/*DcETHR* as the upstream regulator of JH signaling during the fecundity improvement of *D. citri* and *C*Las proliferation. Furthermore, qRT‐PCR results indicated that *DcECR* knockdown significantly inhibited the mRNA expression of *DcJHAMT* and *DcMet* (Figure 6H). Treatment with JHA notably decreased the relative expression level of *DcECR*, while dsDcMet treatment increased the transcript level of *DcECR* (Figure 6I). These findings suggest that 20E and its receptor *DcECR* may stimulate JH signaling via *DcETH*/*DcETHR* signaling, with excessive JH levels possibly suppressing 20E signaling.

## 3 Discussion

*C*Las multiplies inside *D. citri* using a complex internal circulation system that enhances its effectiveness.^[^ ^28^ ^]^ The striking increase in the female reproductive capacity of *C*Las+ *D. citri* undoubtedly exacerbates the occurrence and spread of HLB in the field.^[^ ^7^ ^]^ Investigating how *C*Las manipulates the host endocrine hormone network to boost the fecundity of citrus psyllids at the molecular level may yield novel insights for the effective prevention and control of citrus HLB. ETH, a neuroendocrine hormone, plays a dual role in insect development. It not only contributes to molting during the larval stages but also governs reproduction of adult females.^[^ ^14^ ^]^ For example, the ETH system is essential for the female reproductive success in *Mythimna separata* Walker (Lepidoptera: Noctuidae).^[^ ^29^ ^]^

In this study, we conducted the first analysis of the characteristics of DcETH. The mature peptide sequences of DcETH were relatively conserved when compared analogues from selected species, particularly with the sequences from hemipteran insects (Figure S1, Supporting Information). Our findings found that *C*Las infection markedly increased the expression of *DcETH* in the ovaries of infected females compared to the *C*Las‐ controls, suggesting a potential role for *DcETH* in the molecular interplay between *C*Las and *D. citri*. Manipulating *DcETH* expression increased energy metabolism and various reproductive parameters of *C*Las+ psyllids but also led to a significantly reduction in *C*Las levels within the ovaries (Figure 1). These results deepen our understanding of ETH's function, which not only governs insect reproductive development and response to stress, but also plays a role in the interaction between host insects and pathogens.^[^ ^14^, ^19^, ^29^ ^]^ Furthermore, our study conclusively identified *DcETHR* as the direct receptor of *DcETH* in *D. citri* through the use of in vitro calcium imaging and in vivo RNAi experiments (Figure 1). Consistent with the effects of RNAi on levels of *DcETH*, interference with *DcETHR* led to decreased mRNA expression of two *DcVg* and its receptor *DcVgR*, reductions in TG content, lipid droplet size, ovarian development, egg production, and *C*Las levels in *C*Las+ psyllids (Figure 2). Consequently, we posit that ETH signaling facilitates *C*Las proliferation by affecting the storage of energy substances and the transport of vitellogenin proteins via *DcVgR*. While the function of ETH/ETHR in insect reproductive processes has been well elucidated, the molecular regulatory mechanism of ETHR remain elusive.

miRNAs, small non‐coding RNAs, play a crucial role in regulating reproductive processes in insects and in the insect response to pathogens.^[^ ^30^, ^31^ ^]^ For instance, the miR‐2 cluster collectively targets *Notch* and regulates oogenesis in *Locusta migratoria* (L.) (Orthoptera: Acrididae),^[^ ^32^ ^]^ and miR‐277 targets insulin‐like peptide to govern ovarian development in *Aedes aegypti* L. (Diptera: Culicidae).^[^ ^24^ ^]^ In the context of promoting pathogen proliferation, *C*Las infection boosts energy metabolism and ovarian development of *D. citri* by influencing host miR‐275 and miR‐34.^[^ ^13^, ^33^ ^]^ In this study, we found that miR‐210, which was down‐regulated by *C*Las infection, targeted *DcETHR* resulting in the increased fecundity of *C*Las+ *D. citri* (Figures 3, 4). Further analysis indicated that a decrease in *Dicer‐1* mRNA levels in *C*Las+ females compared to *C*Las‐ individuals, while the expression levels of *Ago‐1* remained consistent between *C*Las+ and *C*Las‐ females. This suggested that *C*Las infection diminishes miR‐210 expression by influencing the activity of the ribonuclease enzyme Dicer‐1. Following miR‐210 agomir administration, *C*Las+ females exhibited reduced TG contents, smaller lipid droplet sizes, delayed ovarian development, decreased egg production, and reduced *C*Las levels compared to agomir‐NC treatments. These findings represent the first report on the post‐transcription regulation of *ETHR* in insects. Further elucidating the regulatory network involving miR‐275, miR‐34, and miR‐210 in the fecundity enhancement of *C*Las+ *D. citri* promises to be an intriguing area for future investigation.

The release of ETH from Inka cells is intricately regulated by modulating concentrations of the ecdysteroid, 20E.^[^ ^34^ ^]^ 20E plays a direct role in regulating the expression of both ETH and its receptor ETHR during ecdysis and reproductive process.^[^ ^19^, ^35^ ^]^ Our findings revealed a significant decrease in 20E levels, and reduction in the mRNA expression of the 20E biosynthesis genes and receptors, upon *C*Las infection. Hence, we conclude that *C*Las infection decreases 20E titer in *D. citri* ovaries by modulating the mRNA expression of genes involved in 20E biosynthesis. Treatment with 20E in vitro notably increased the expression of *DcETH* and *DcETHR*, while knockdown of the 20E receptor led to a dramatic inhibition of their expression. Knapp and Sun noted that in *Drosophila*, 20E produced in follicle cells activates EcR.B2 to initiate maturation of follicles, suggesting that 20E is not exclusively derived from the head but also from the ovary itself.^[^ ^36^ ^]^ Exogenous 20E treatments could potentially stimulate ETH synthesis, leading to the upregulation of *DcETH* and subsequent activation of *DcETHR* in the ovaries independently of the head. Subsequent results of RNAi demonstrated that knockdown of *DcECR* resulted in similar phenotypic defects in energy metabolism, fecundity, and *C*Las titres as observed with *DcETHR* interference. Interestingly, treatment with DcETH effectively rescued these defects caused by *DcECR* knockdown (Figure 5). These data suggest that 20E signaling acts upstream of *DcETH*/*DcETHR* in enhancing fecundity in *C*Las+ *D. citri* and promoting *C*Las proliferation. Prior research has indicated that ETH also contributes to JH biosynthesis by activating the key enzyme, JHAMT, of the JH biosynthetic pathway, a process dependent on ETHR expression.^[^ ^37^ ^]^ In our study, RNAi‐mediated knockdown of *DcETHR* markedly reduced the expression of *DcJHAMT* and *DcMet* (Figure 6C,D). Treatment with JHA completely rescued the phenotypic defects in energy metabolism, fecundity, and *C*Las levels caused by *DcETHR* knockdown, suggesting that ETH signaling functions upstream of JH signaling in enhancing fecundity in *C*Las+ *D. citri* and promoting *C*Las proliferation.

**Figure 7:**
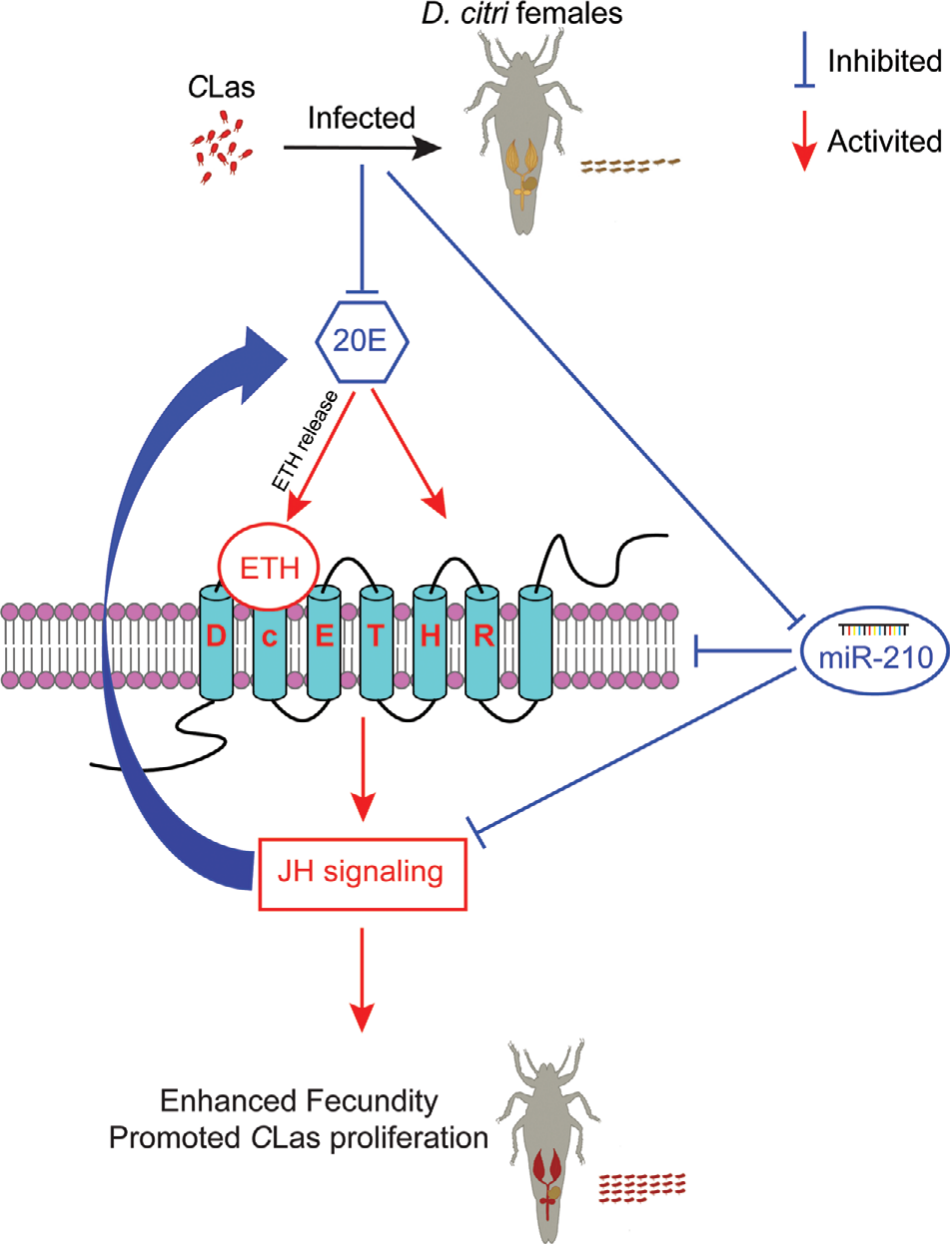
A molecular model for host miR‐210 and ETH signaling mediate the enhanced fecundity of *C*Las‐infected *D. citri* females and *C*Las proliferation.

In summary, our study reveals an endocrine network involving in 20E‐ETH‐JH in the mutualistic interaction between *C*Las and *D. citri* within the ovaries. We present a schematic overview of this novel mechanism in **Figure** **7**. In *C*Las+ *D. citri*, *C*Las initially reduces 20E levels, thereby alleviating the inhibition of ETH release. Subsequently upregulation of ETH promotes the expression of its receptor, *DcETHR*, further activating the JH signaling pathway. This cascade ultimately enhances energy metabolism, leading to increased TG and lipid droplet contents, thereby boosting female fecundity and facilitating *C*Las proliferation. Additionally, miR‐210 targets *DcETHR* and is involved in the regulation of the endocrine network responsible for the higher fecundity *C*Las+ *D. citri* and *C*Las proliferation. In order to better manage Huanglongbing in the field, future researches will focus on the following directions: (1) RNA biopesticides targeting *DcETH* and *DcETHR* through RNAi can be prepared to block the ovarian development of *D. citri*, reduce their population size, and thus prevent the spread of Huanglongbing. (2) By introducing miRNA‐210 or dsRNAs of *DcETH* and *DcETHR* into citrus plants to construct transgenic citrus, the ovarian development and population size of feeding psyllids can be affected, ultimately controlling the field transmission of Huanglongbing. (3) Exploring the involvement of transboundary miRNAs and plant‐derived hormones in enhancing fecundity in *C*Las+ *D. citri* and *C*Las proliferation.

## 4 Experimental Section

### Insects and Host Plants

The *D. citri* colonies, categorized as either *C*Las‐negative (*C*Las‐) or *C*Las‐positive (*C*Las+), were originally sourced from citrus trees in Zhanjiang city, Guangdong province, during the summer of 2020, aligning with the *C*Las status of the trees. Subsequently, the *C*Las‐negative colonies were consistently reared on healthy, CLas‐free lemon plants (*Citrus* × *limon* (L.) Osbeck), while the *C*Las+ colonies were maintained on *C*Las‐infected lemon plants.

These two cohorts were housed in separate incubators maintained under uniform conditions: a temperature of 26 ± 1 °C, relative humidity of 65 ± 5%, and a light‐dark cycle of 14 to 10 h. The *C*Las‐ and *C*Las+ lemon plants were cultivated in different glasshouses. Regular monthly monitoring of *C*Las infection levels in both the lemon plants and psyllids was conducted using quantitative polymerase chain reaction (qPCR) as previously described.^[^ ^13^ ^]^

### Sequence Analysis and Molecular Docking

The mRNA sequences of *DcETH* (GenBank accession number: MG550169.1) and *DcETHR* (GenBank accession number: MG550195.1) were retrieved from the NCBI database. The physicochemical characteristics of *DcETH* and *DcETHR* were assessed utilizing the ProtParam tool available online (http://web.expasy.org/protparam/). To predict potential transmembrane domains of *DcETHR*, the SMART software (Simple Modular Architecture Research Tool) was employed. Homologous protein sequences from diverse insect species were identified through a BLASTP search within the NCBI database. Amino acid sequences alignments for *DcETH* and *DcETHR* with their homologs were conducted using DNAman software. Subsequently, a phylogenetic analysis was carried out employing the neighbor‐joining (NJ) method in MEGA10.1.8 software. The tertiary structures of *DcETH* and *DcETHR* were predicted using the Phyre^2^ online server (http://www.sbg.bio.ic.ac.uk/phyre2/html/page.cgi?id=index) and further refined with PyMOL‐v1.3r1 software.^[^ ^38^ ^]^

### Amplification Conditions of Semi‐Quantitative RT‐PCR and QRT‐PCR

Female adults that had recently emerged (within 24 h) were labeled as 1 day after eclosion (DAE). To represent various developmental stages, ovaries were collected from females 4, 8, and 12 DAE, both from *C*Las‐ and *C*Las+ adults. Additionally, for spatial expression analysis, four tissues (head, midgut, ovary, and fat body) were dissected from 12 DAE *C*Las‐ females.

For mRNA quantification, the extraction of total RNA was carried out using TRNzol Universal (Cat# DP424, TIANGEN, Beijing, China). Subsequently, first‐strand cDNA was synthesized following the manufacturer's protocol using the PrimeScript RT reagent kit with gDNA Eraser (Cat# RR047A, Takara, Kyoto, Japan). For semi‐quantitative RT‐PCR reactions, the PCR cycles were determined by a gradient increase from 20 to 30 cycles. qRT‐PCR was conducted utilizing the TB Green Premix Ex TaqTM II (RR820, Takara, Beijing, China) in a 20 µL reaction mixture on an ABI PRISM 7500 Real‐Time System (Applied Biosystems, Foster City, CA, USA). For determining the *C*Las titer in *C*Las+ psyllids, qRT‐PCR was employed following the previous method.^[^ ^13^ ^]^ To analyze miRNA expression, RNA enrichment was conducted using the miRcute miRNA Isolation Kit (DP501, TIANGEN, Beijing, China). Next, mature miRNA was synthesized employing the miRcute Plus miRNA First‐Strand cDNA Kit (KR211, TIANGEN, Beijing, China). Quantification analysis of miRNA was carried out utilizing the miRcute Plus miRNA qPCR Kit (SYBR Green) (FP411, TIANGEN, Beijing, China). The housekeeping genes, beta‐actin (*Dcβ‐ACT*, XM_026823249.1) and U6 snRNA, were utilized for normalizing the expression levels of mRNA and miRNA in *D. citri*. For each primer pair, a standard curve was established through serial dilutions of cDNA to determine both the amplification efficiency and CT value. The primers utilized in this study are detailed in Table S1. qRT‐PCR data were quantified using the 2^−ΔΔCT^ method, where ΔΔCT is equal to ΔCT_treated sample_ ‐ΔCT_control_.^[^ ^39^ ^]^

### Experimental Procedures for RNAi and miRNA Treatment

The synthesis of double‐stranded RNAs (dsRNAs) targeting *DcETH*, *DcETHR*, and other genes was carried out using the MEGAscript RNAi kit (AM1626, Ambion, California, USA), followed by purification with the MEGAclear columns (Ambion) and eluted with diethyl pyrocarbonate (DEPC)‐treated nuclease‐free water. For RNAi experiments, EGFP dsRNA was utilized as the negative control. The synthesis of miRNA agomir and antagomir was conducted at GenePharma (Shanghai, China), and the manufacturer supplied the negative control for both agomir and antagomir.

Following the previously described methodology, RNAi and miRNA treatments were conducted by administering dsRNA or miRNA antagomir/agomir through an artificial diet.^[^ ^13^, ^40^ ^]^ Specifically, twenty female psyllids 6 DAE were housed in a glass cylinder (25 × 75 mm) sealed with two stretched sections of Parafilm. A 200 µL solution of 20% (w/v) sucrose containing dsRNA was delivered between the Parafilm. The final concentrations of dsDcETH and dsDcETHR were set at 500 ng µL^−1^ each. Following a 48‐h feeding period, the treated females underwent a series of assays. Ovaries were dissected for the analysis of RNAi efficiency using qRT‐PCR. Parameters related to reproduction, specifically fecundity, were documented. The quantification of *C*Las titers in the ovaries was performed. Ovary morphology was visualized using an Ultra‐Depth 3D Microscope (VHX‐500), while fat bodies were stained with Nile red. Levels of triacylglycerol (TG) were assessed. To ensure robustness and reliability, all experiments were replicated at least three times, with each replication comprising 15–20 pairs of *D. citri*.

### Nile Red Staining and Triglycerides Levels Quantification

To visualize lipid droplets, Nile red (SL0201, Coolaber, Beijing, China) was employed for staining the fat bodies following the standard procedure. Initially, fat bodies were isolated from female specimens of different experimental groups, fixed in 4% paraformaldehyde at room temperature, and rinsed with 1 × phosphate buffered saline (PBS) on multiple occasions. Subsequently, the tissues were immersed in a solution containing Nile red (0.1 µg µL^−1^) and DAPI (0.05 µg µL^−1^) for 30 min, followed by two washes with 1× PBS. The visualization and capture of fluorescence images were carried out using Leica SP8 confocal microscopy (Weztlar, Germany).

For the quantification of TG levels, a Triglycerides Colorimetric Assay (Cayman) was performed in accordance with the manufacturer's guidelines (A110‐1‐1, Nanjing Jiancheng, China). In brief, fat bodies from 30 females were homogenized in 100 µL of Diluent Assay Reagent, followed by the incubation of 10 µL of the resulting supernatant in the Enzyme Mixture solution. The TG concentrations were normalized and quantified concerning the protein levels in the supernatant, as determined by a BCA protein assay (Thermo Fisher Scientific). Each experimental treatment underwent three independent biological replicates, with each replicate comprising three technical replicates.

### Fluorescence in Situ Hybridization (FISH) for CLas Localization

To investigate the spatial distribution of *DcETHR*, miR‐210, and *C*Las in the ovary, FISH was conducted utilizing specific probes (Table S1) as detailed in a previous study.^[^ ^13^ ^]^ Initially, the ovaries were carefully dissected and fixed in Carnoy's fixative for 24 h at room temperature. Following fixation, the ovaries were rinsed with 100% ethanol, decolorization in 6% H_2_O_2_ in ethanol, and pre‐hybridization performed thrice using the hybridization buffer (comprising 20 mm Tris‐HCl, pH 8.0; 0.9 m NaCl; 0.01% sodium dodecyl sulfate; 30% formamide) without the inclusion of the probe, which are all conducted in darkness. Subsequently, the samples were subjected to hybridization in the dark for a minimum of 12 h in a hybridization buffer containing 1 µM fluorescent probe. Following three washes with PBST, cell nuclei were stained with DAPI (1 µg mL^−1^) for 15 min, followed by rinsing with PBST for 15 min. Finally, the stained samples were mounted on a microscope slide using a cover slip and visualized using Leica SP8 confocal microscopy (Weztlar, Germany). To validate the specificity of the *C*Las signal, *C*Las‐ ovaries or samples lacking the probe were utilized as negative controls.

### Ca^2+^ Imaging and Fluorescence Detection of Fluo‐4 AM

The open reading frame (ORF) of *DcETHR* was amplified via PCR utilizing specific primers detailed in Table S1. Subsequently, the amplified sequence was integrated into the pcDNA3.1(+)‐mCherry vector to generate the recombinant DcETHR‐pcDNA3.1‐mCherry vector using the pEASY‐Basic Seamless Cloning and Assembly Kit (Cat# CU201, TransGen, Beijing, China). The recombinant vector was then transfected into HEK293T cells employing TransIntro EL Transfection Reagent (Cat# FT201, TransGen, Beijing, China), and the transfected cells were cultivated at 37 °C for a period ranging from 20 to 40 h. Following this, the transiently transfected cells were subjected to staining by incubating them in 500 µL of Fluo‐4 AM (Cat# S1061, Beyotime, Shanghai, China) for 30 min at 37 °C. Subsequently, the cells were washed thrice with Hank's Balanced Salt Solution. The treated cells were exposed to empty vector or mature peptide of DcETH for further experimentation. These cells were utilized for calcium ion (Ca^2+^) imaging employing a Leica SP8 confocal microscopy (Weztlar, Germany) or for the fluorescence detection of Fluo‐4 AM using a Molecular Devices i3x microplate reader (San Jose, USA).

### Prediction and Validation of MiRNAs Targeting DcETHR

The identification of miRNAs targeting *DcETHR* was conducted by the service provider LC Science through the analysis of the 3′UTR or coding sequence (CDS) using two distinct software tools, namely miRanda (http://www.microrna.org) and TargetScan (https://www.targetscan.org/vert_72/), following the default parameters.^[^ ^41^, ^42^ ^]^ For miRanda software, alignment scores of 145 and ‐15 kcal mol^−1^ were utilized as thresholds. In the case of TargetScan, a context score percentile greater than 50 was employed to assess the relationship between the miRNA and potential targets. The miRNAs that were predicted by both methods were selected for further analysis.

Subsequently, the following three distinct assays were carried out to confirm the target relationship. (1) For in vitro luciferase activity assays, a 3558 bp segment of the CDS sequence containing the predicted binding sites for miR‐210 in *DcETHR* was cloned downstream of the luciferase gene in the pmirGLO vector (Promega, Wisconsin, USA) to create the recombinant plasmid DcETHR‐CDS‐pmirGLO. The mutated CDS sequence was also amplified and inserted into the pmirGLO vector to generate the DcETHR‐CDS‐mutant‐pmirGLO plasmid. HEK293T cells were transfected with either the DcETHR‐CDS‐pmirGLO plasmid (≈500 ng) or the DcETHR‐CDS‐mutant‐pmirGLO plasmid (≈500 ng), along with 275 nM of agomir‐miRNA or agomir‐NC, using the Calcium Phosphate Cell Transfection Kit (Cat# C0508, Beyotime, Nanjing, China). The activity of the luciferase enzymes was assessed 24 h post‐transfection following the manufacturer's guidelines for the Dual‐Luciferase Reporter Assay System (Cat# E1910, Promega, Wisconsin, USA). Each transfection was replicated nine times to obtain statistically robust data. (2) For in vivo RNA‐binding protein immunoprecipitation assays (RIP), females at 8 DAE were exposed to either agomir‐210 or agomir‐NC for 24 h and then subjected to RIP assay 10 h later following the protocol of Magna RIP Kit (Cat# 17–704, Merck, Millipore, Germany). The dissected ovaries were homogenized in RIP lysis buffer, and overnight storage at −80 °C. Magnetic beads were then combined with Ago‐1 antibody or IgG antibody to form bead‐antibody complexes. Subsequent steps involved sample division, incubation, RNA release, and qRT‐PCR quantification of *DcETHR* and miR‐210 levels. “Input” samples and IgG controls were utilized for normalization and validation of RNA‐protein interactions, with three replicates conducted per treatment. (3) To assess the impact of miR‐210 on *DcETHR* expression, females at 6 DAE were fed with agomir‐210 (1 µM) or agomir‐NC (1 µM), and collected at 48 h after treatments for expression analysis of *DcETHR* and miR‐210 using qRT‐PCR.

### Experiments Involving Juvenile Hormone and Ecdysone Treatments

Ovarian ecdysone (20E) levels were assessed using the Insect Ecdysone ELISA kit (ml057449, Shanghai Meilian Biotechnology, China) following the manufacturer's guidelines. Female psyllids were administered a synthetic JH analogue (methoprene, JHA) (15 mg mL^−1^; Product No. 33375, Sigma‐Aldrich, MO, USA) or 20E (15mg mL^−1^; A506554, Sangon Biotech., China) for a duration of 48 h. Dimethyl sulphoxide (DMSO) served as the control when psyllids were exposed to the hormone treatments. For each replicate, twenty ovaries were combined, and a total of three replicates were conducted.

### Statistical Analysis

GraphPad Prism 8.0 software was utilized for generating figures, while IBM SPSS Statistics 20.0 was employed for statistical analyses. The data were presented as means ± standard errors (SE) with three or nine independent biological replicates. For mRNA expression analysis of *DcETH* and *DcETHR*, pairwise comparisons between treatments and controls were conducted using Student's *t*‐test to identify statistically significant variances (*p < 0.05, **p < 0.01, ***p < 0.001). For statistical analyses of TG contents, fecundity, relative *C*Las titer, and relative luciferase activity, a one‐way ANOVA was executed followed by Tukey's HSD multiple comparison test, where varying letters indicated statistical significance (*p* < 0.05).

## Conflict of Interest

The authors declare no conflict of interest.

## Author Contributions

X.N. and B.W. contributed equally to this work. S.Z. and X.N. designed research; S.Z., B.W., and X.N. performed research; P.H., G.A.C.B., S.T., W.Y., Y.C., and Y.H. contributed new analytic tools; S.Z., B.W., and X.N. analyzed data; S.Z., X.N., P.H., and G.A.C.B. wrote the paper; S.Z., X.N., and Y.H. provided the fund.

## Supporting information



Supporting Information

## Data Availability

The published article includes all data generated or analyzed during this study. The full sequences were submitted to NCBI, accession number: MG550169.1 for *DcETH*, MG550195.1 for *DcETHR*.
