# Supplementary material for: Neuropeptide Ecdysis‐Triggering Hormone and Its Receptor Mediate the Fecundity Improvement of ‘Candidatus Liberibacter Asiaticus’‐Infected Diaphorina citri Females and CLas Proliferation
Source: Adv Sci (Weinh). 2025 Mar 20;12(18):2412384. doi: 10.1002/advs.202412384 (PMC12079412; doi:10.1002/advs.202412384)
Supplement: Supplementary file 1 — Supporting Information [file ADVS-12-2412384-s001.docx]

Supporting Information

**Neuropeptide Ecdysis-Triggering Hormone and Its Receptor Mediate the Fecundity Improvement of ‘*Candidatus* Liberibacter asiaticus’-Infected *Diaphorina citri* Females and *C*Las Proliferation**

*Songdou Zhang*,* *Bo Wang*, *Paul Holford*, *George Andrew Charles Beattie*, *Shijian Tan*, *Weiwei Yuan*, *Yijing Cen*, *Yurong He*, and *Xiaoge Nian**

**
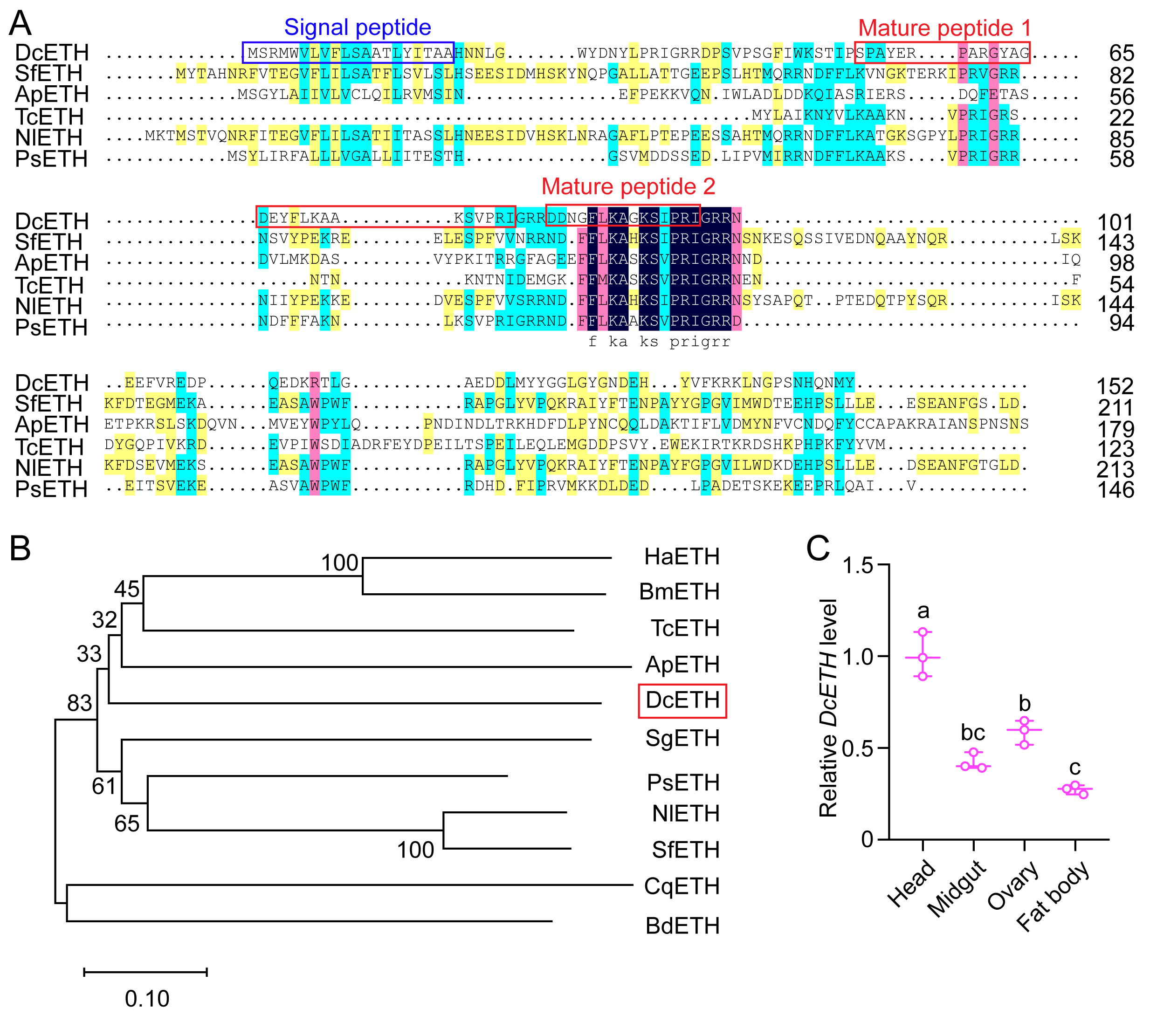
**

**Figure S1.** Sequence analysis and tissue expression of *DcETH*. A) Alignment of amino acid sequence of DcETH with homologs from other five insect species. Black indicates 100% identity, red denotes 75% identity, cyan signifies less than 50% identity, and yellow represents less than 33% identity. The signal peptide is highlighted in a blue box and the two mature peptides are marked with red boxes. DcETH (*D. citri*, AWT50603.1), SfETH (*Sogatella furcifera*, QDZ26132.1), ApETH (*Acyrthosiphon pisum*, NP_001156684.1), TcETH (*Tribolium castaneum*, EFA07492.2), NlETH (*Nilaparvata lugens*, BAO00949.1), PsETH (*Plautia stali*, BAV78804.1). B) Phylogenetic analysis depicting the relationship between DcETH and homologous proteins from ten other insect species. HaETH (*Helicoverpa armigera*, WGD18913.1), BmETH (*Bombyx mori*, NP_001165743.1), SgETH (*Schistocerca gregaria*, UGX04191.1), CqETH (*Culex quinquefasciatus*, EDS43683.1), BdETH (*Bactrocera dorsalis*, AVA17335.1). Accession numbers for the other six homologous proteins were the same as in Figure S1A. C) Relative mRNA expression of *DcETH* in different tissues of *C*Las- females. The data for each tissue consists of three independent replicates, with at least 30 females per replicate. Differences among the means were determined by one-way ANOVA followed by Duncan’s multiple range tests. Different letters above the data points indicate significant differences between treatments at *P*<0.05.


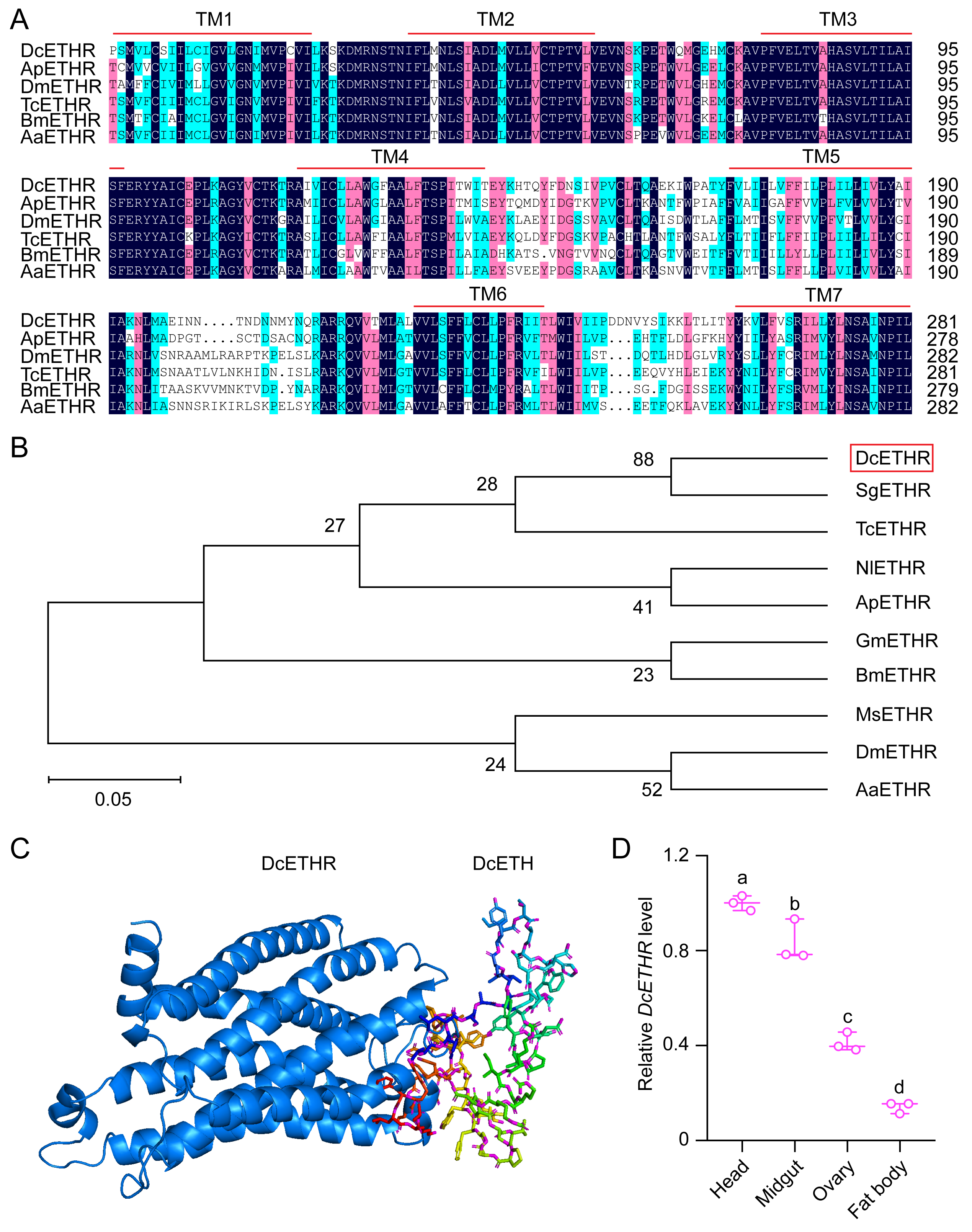


**Figure S2.** Sequence analysis and tissue expression of *DcETHR*. A) The transmembrane domain amino acid sequences of DcETHR were aligned with analogs from five other insect species. In the alignment, black denotes 100% identity, red indicates 75% identity, and cyan signifies less than 50% identity. Transmembrane domains from TM1 to TM7 are delineated by red horizontal lines. DcETHR (*D. citri*, AWT50629.1), ApETHR (*Acyrthosiphon pisum*, DAA64797.1), DmETHR (*Drosophila melanogaster*, NP_650960.2), TcETHR (*Tribolium castaneum*, NP_001076792.1), BmETHR (*Bombyx mori*, NP_001127741.1), AaETHR (*Aedes aegypti*, ABI93273.1). B) Phylogenetic analysis of DcETHR with homologous proteins from nine other insect species. MsETHR (*Manduca sexta*, AAX19163.1), GmETHR (*Grapholita molesta*, QPZ46771.1), SgETHR (*Schistocerca gregaria*, ARK38543.1), NlETHR (*Nilaparvata lugens*, XP_039284950.1). Accession numbers of the other six homologous proteins were the same as in Figure S2A. C) Predicted tertiary protein structure of DcETHR and its binding with DcETH. D) The tissue expression pattern of *DcETHR* in *C*Las- females. The data for each tissue consists of three independent replicates, with at least 30 females per replicate. Differences among the means were determined by one-way ANOVA followed by Duncan’s multiple range tests. Different letters above the data points indicate significant differences between treatments at *P*<0.05.


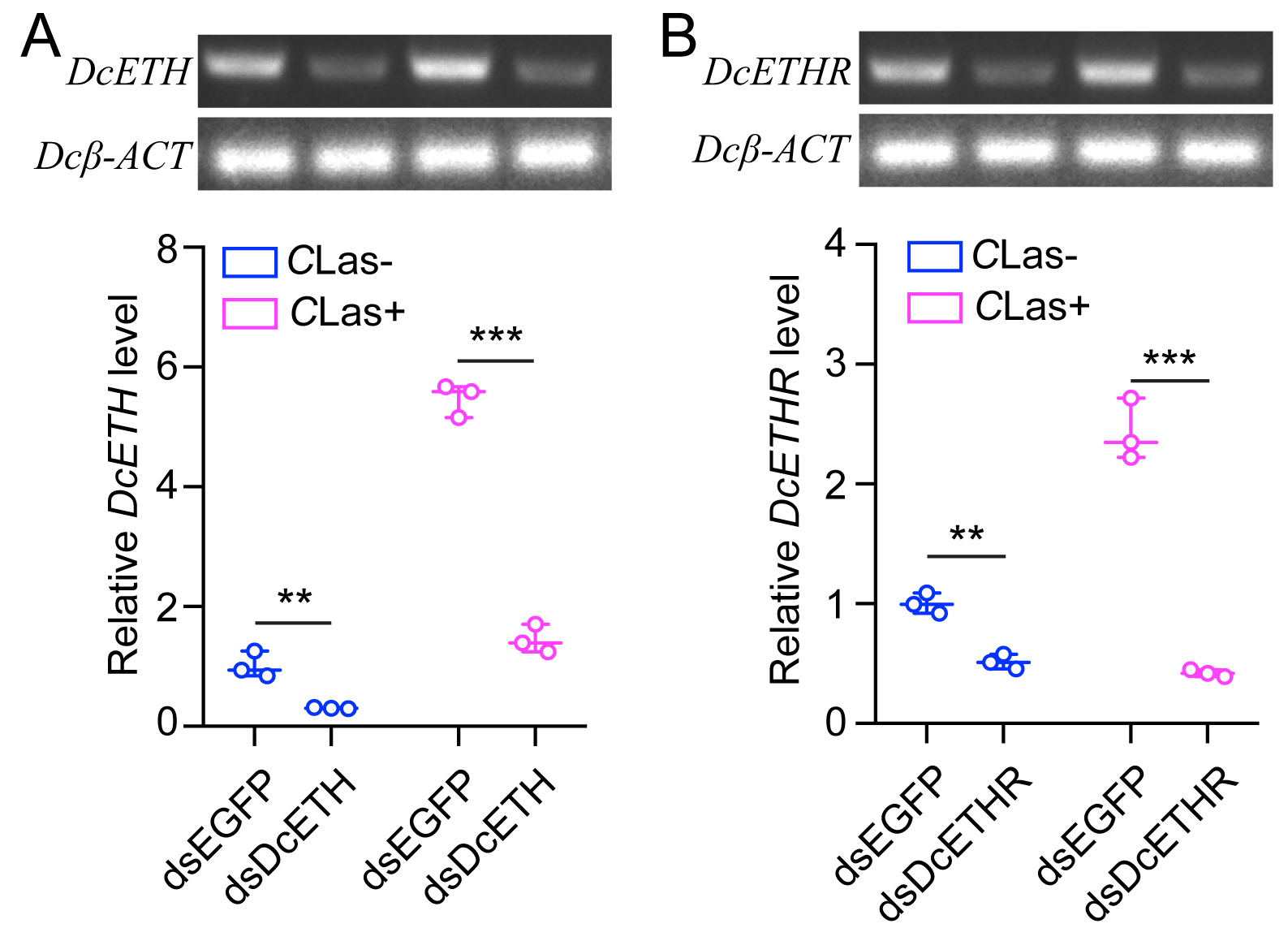


**Figure S3.** RNAi efficiency of *DcETH* and *DcETHR* in *C*Las+ and *C*Las- females after dsRNA feeding at 48 h by semi-quantitative RT-PCR and qRT-PCR. Statistically significance between different groups was determined using pair-wise Student’s *t*-test, with significance levels denoted by ** (*p*<0.01) and *** (*p*<0.001).


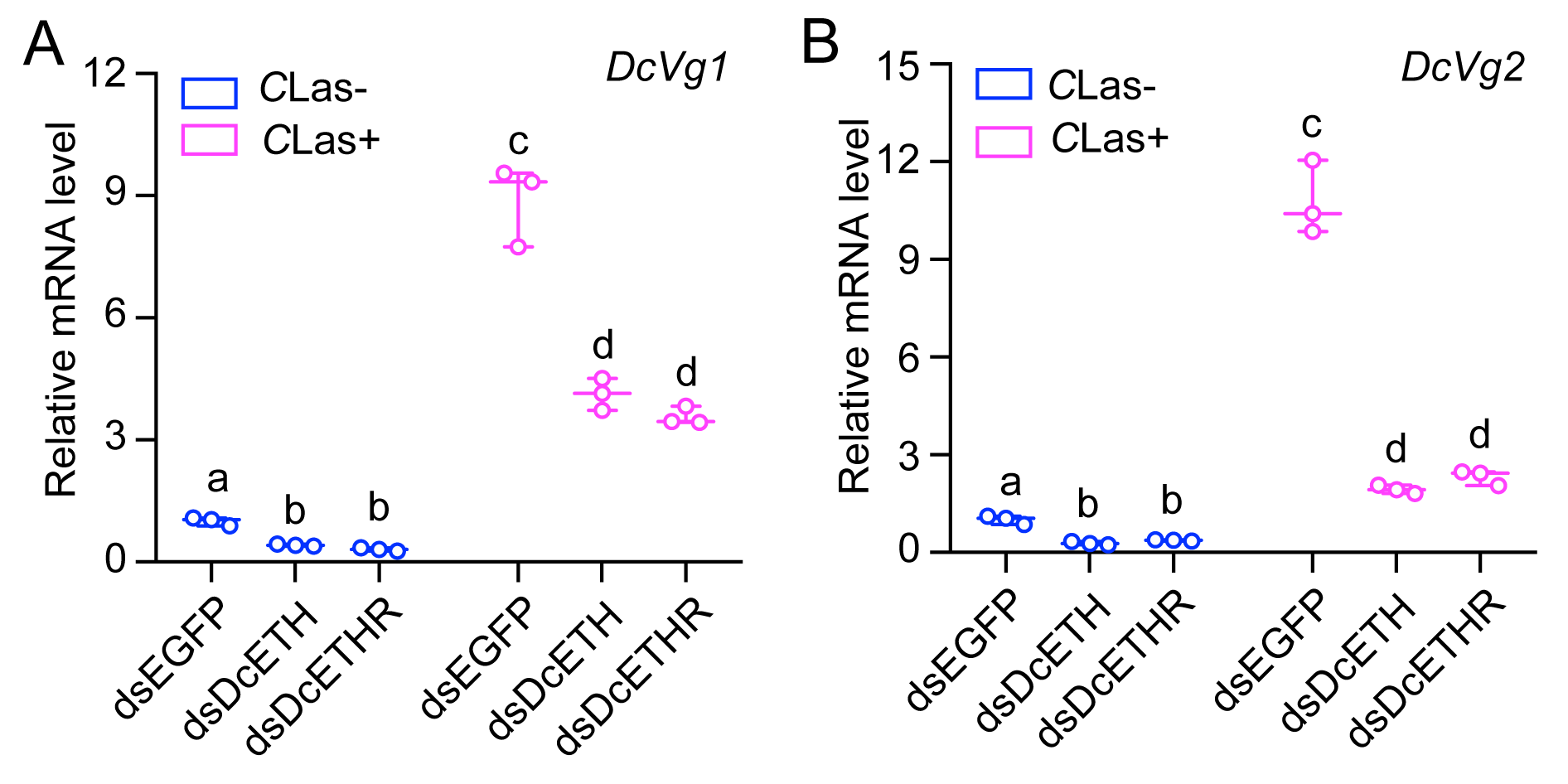


**Figure S4.** The impact of *DcETH* and *DcETHR* knockdown on the mRNA expression of *DcVg1* and *DcVg2* in both *C*Las+ or *C*Las- females. The data are mean ± SEs with three independent biological replicates. The data were subjected to ANOVA followed by a Tukey’s Honest Significant Difference tests. Different letters above the bars denote statistically significant differences at *p*<0.05.


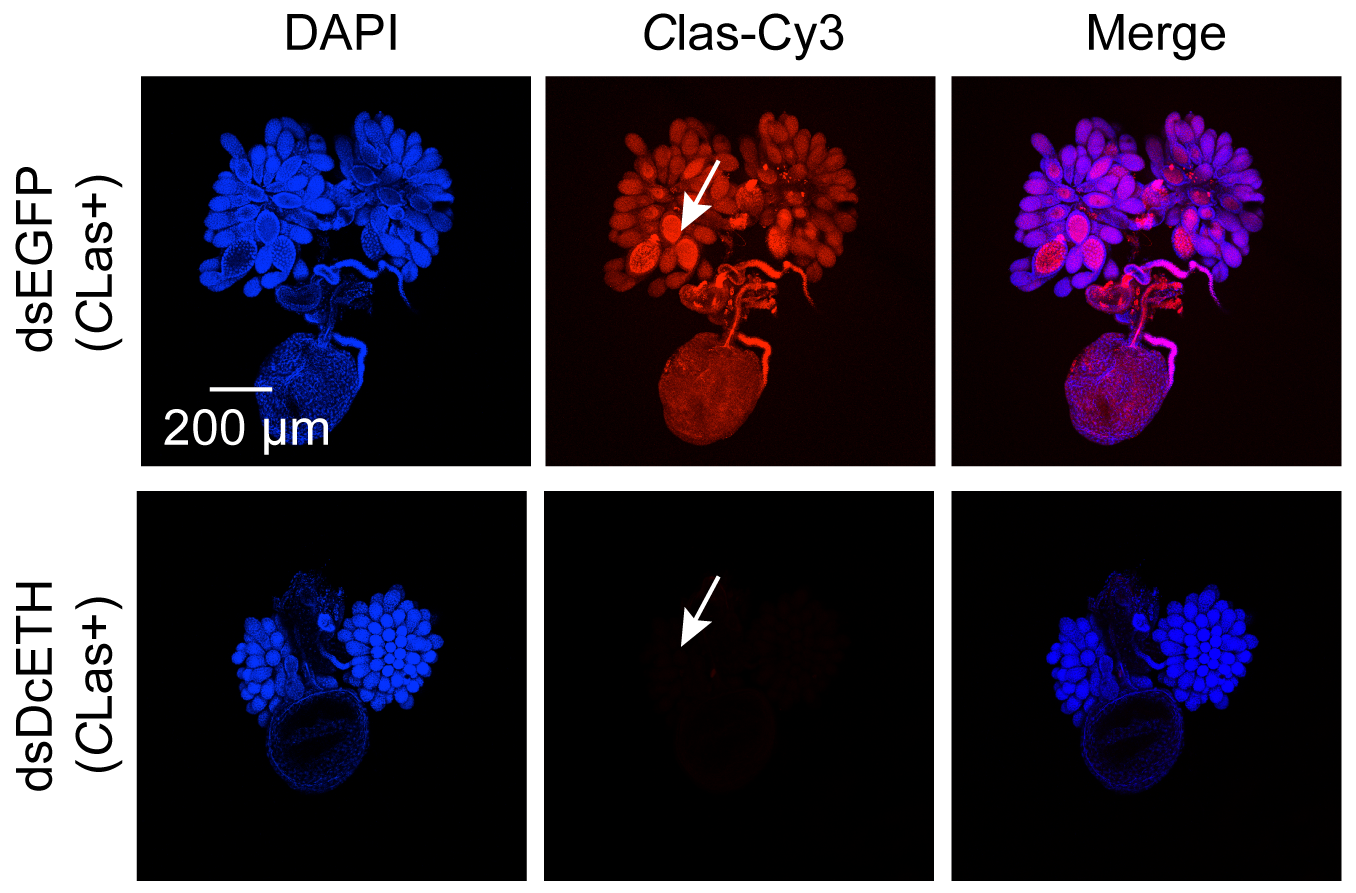


**Figure S5.** Effect of *DcETH* knockdown on *C*Las levels in *C*Las+ females by FISH. The scale bar for ovary is 200 μm. DAPI staining was used to visualize cell nuclei in blue. *C*Las-Cy3 labeling was used to detect the *C*Las signal and visualized in red. The merged image combined the imaging of DAPI and *C*Las-Cy3 signals.


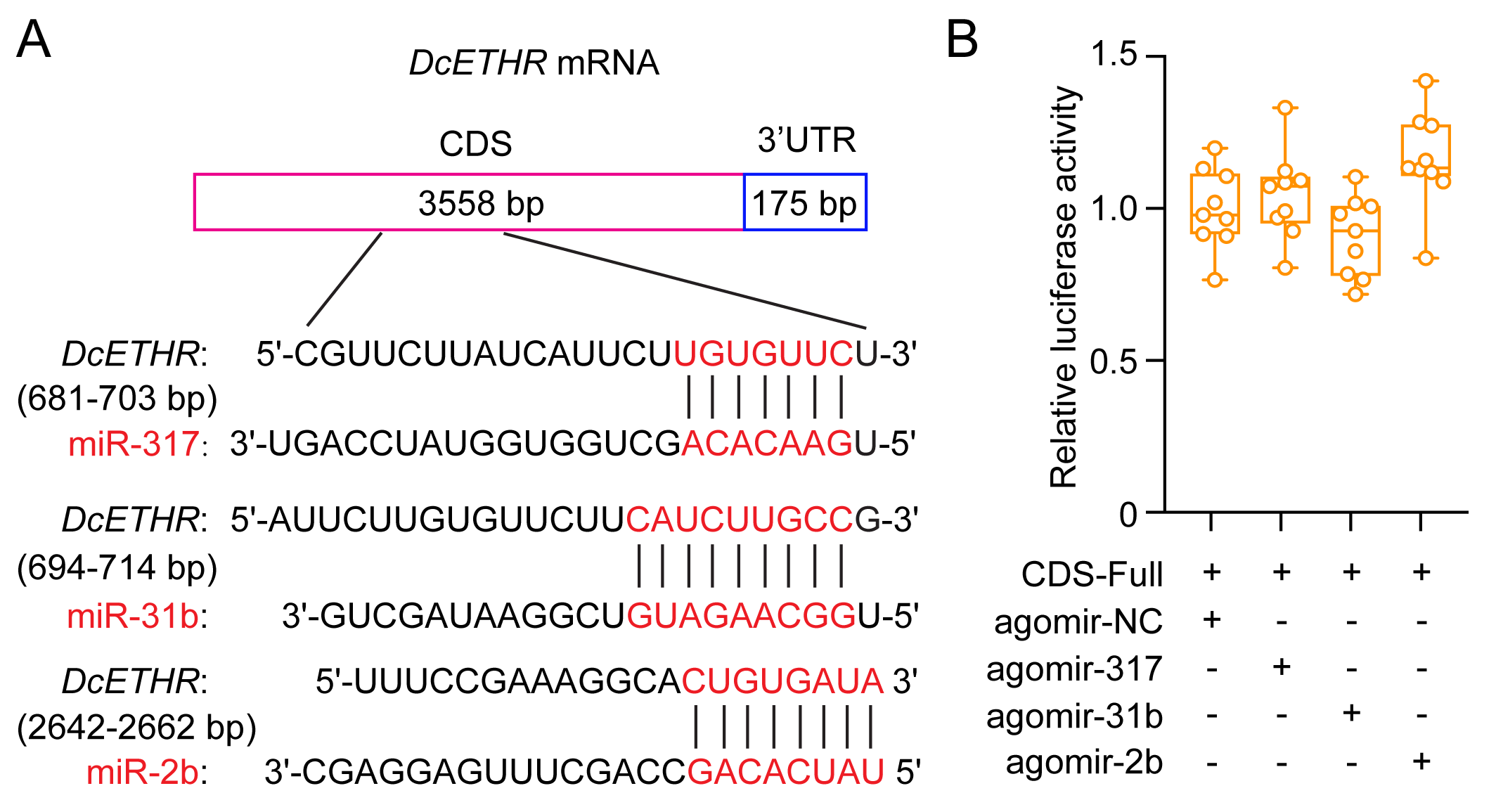


**Figure S6.** Experimental validation of the interactions between *DcETHR* and other three specific miRNAs. A) Predicted binding sites of miR-317, miR-31b, and miR-2b in the CDS of *DcETHR* determined using miRanda and Targetscan. B) Dual luciferase reporter assays conducted *in vitro* to validate the interactions between *DcETHR* and three miRNAs (miR-317, miR-31b, miR-2b). Results are presented as means ± SEs with nine biological replicates. The data were subjected to ANOVA followed by a Tukey’s Honest Significant Difference tests. Different letters above the bars denote statistically significant differences at *p*<0.05.


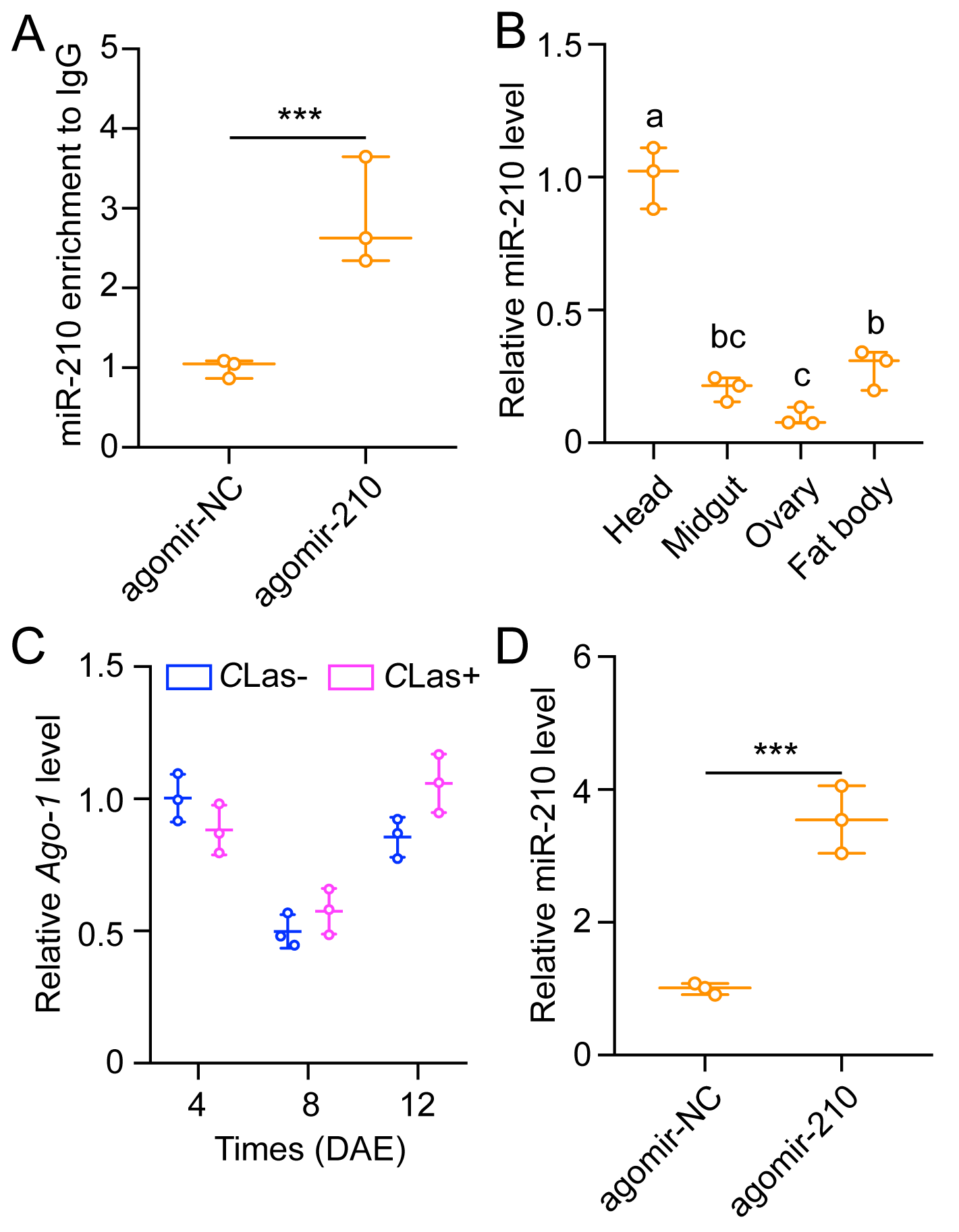


**Figure S7.** Expression pattern analysis of *Ago-1* and miR-210. A) Enrichment of miR-210 expression by Ago-1 antibody in the agomir-210 treated group compared to the agomir-NC group. B) miR-210 expression pattern in different tissues of *C*Las- females. C) Influence of *C*Las infection on the mRNA expression of *Ago-1* at 4, 8, and 12 DAE of *C*Las+ females compared to *C*Las- individuals. D) Effect of agomir-210 treatment on miR-210 expression. Data presented as mean ± SEs from three biological replicates. Statistical significance was evaluated using pair-wise Student’s *t*-test, with significance levels denoted by *** (*p*<0.001). Different letters above the bars indicate statistically significant differences (*p*<0.05), determined by ANOVA followed by Tukey’s HSD multiple comparison test using SPSS 20.0 software.


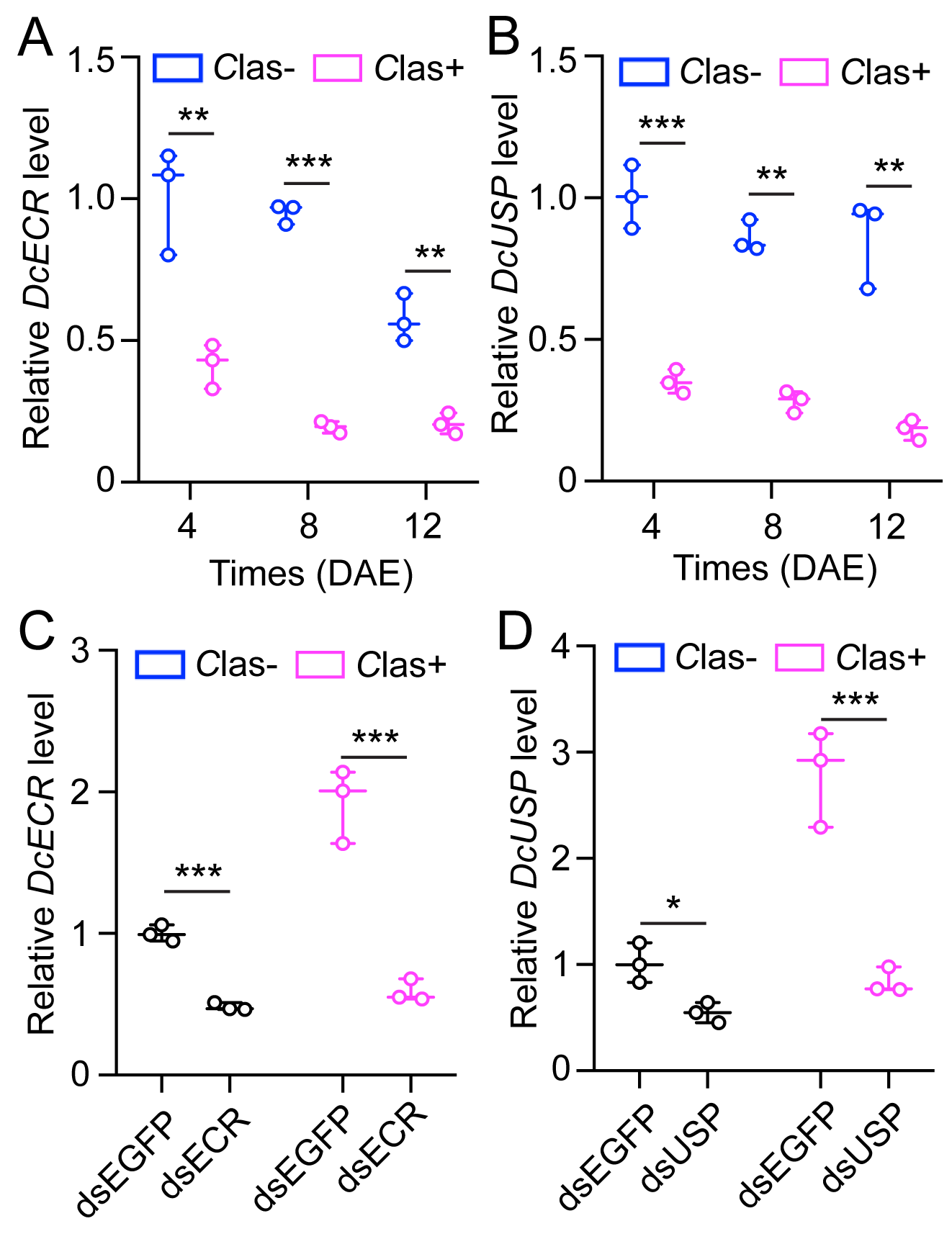


**Figure S8.** Expression pattern analysis of *DcECR* and *DcUSP*. A-B) Influence of *C*Las infection on mRNA expression of *DcECR* and *DcUSP* across various development stages of *C*Las+ females in comparison to *C*Las- individuals. C-D) RNAi efficiency of *DcECR* and *DcUSP* in *C*Las+ and *C*Las- females after dsRNA feeding at 48 h. Data presented as mean ± SEs from three biological replicates. Statistical significance was evaluated using pair-wise Student’s *t*-test, with significance levels denoted by * (*p*<0.05), ** (*p*<0.01), and *** (*p*<0.001).

**Table S1. The primers used in this study.**

| **Gene names and accession number** | **Sequences (5’-3’)** | **Production size** |
| --- | --- | --- |
| *DcETH* (MG550169.1) | qF: ACAACGGATTCCTCAAAGC | 200 bp |
|  | qR: ATTCTGATGATTTGACGGGC |  |
|  | RNAi-F: GATCACtaatacgactcactatagggTGGGCTGGTACGACAACTATC | 192 bp |
|  | RNAi-R: GATCACtaatacgactcactatagggGGAATCCGTTGTCGTCTCTC |  |
| *DcETHR* (MG550195.1) | 3’UTR-F: GACCTTCTCGCACCGTTCTC | 360 bp |
|  | 3’UTR-inner: CGCGGATCCTCCACTAGTGATTTCACTATAGG |  |
|  | qF: AATTCCAGTCCAACCTCATC | 204 bp |
|  | qR: CGTCATTCTCCGAGTTAGAG |  |
|  | RNAi-F: GATCACtaatacgactcactatagggTTGTGTTCTTCATCTTGCCG | 485 bp |
|  | RNAi-R: GATCACtaatacgactcactatagggGGTTTGGCGTTGACTTCTGT |  |
|  | FISH: CGACAUAACUGUGACGGCACUGGUUAAAUCU-FITC |  |
|  | CDS-Full-F: CTAGTTGTTTAAACGAGCTCATGGTGTCCACGACTGTCCAG | 3615 bp |
|  | CDS-Full-R: TGCATGCCTGCAGGTCGACTCTAGATACAAAGGTTTCCTCGTTG |  |
|  | CDS-Mutant-F: CTAGTTGTTTAAACGAGCTCATGGTGTCCACGACTGTCCAG | 3513 bp |
|  | CDS-Mutant-R: TGCATGCCTGCAGGTCGACTCTAGACTTGACGTCATTCTCCGAG |  |
| *Dcβ-ACT* (XM_026823249.1) | qF: TGTTCCAACCTTCCTTCCTG | 109 bp |
|  | qR: GTGTTGGCGTACAGGTCCTT |  |
| *C*Las*16s rRNA* (L22532.1) | qF: TCGAGCGCGTATGCAATACG | 75 bp |
|  | qR: GCGTTATCCCGTAGAAAAAGGTAG |  |
|  | FISH: CATTATCTTCTCCGGCG-Cy3 |  |
| *EGFP* (ACY56286) | RNAi-F: GATCACtaatacgactcactatagggACTCCAGCAGGACCATGTGATC | 596 bp |
|  | RNAi-R: GATCACtaatacgactcactatagggACCTGAAGTTCATCTGCACCAC |  |
| miR-210 | qF: CTGTGCGTGTGACAGCGGCTAT |  |
|  | FISH: ATAGCCGCTGTCACACGCACAAG-Cy3 |  |
|  | agomir: UUUGAUUACGUCACAGACGCACAAG |  |
|  | antagomir: CUUGUGCGUCUGUGACGUAAUCAAA |  |
|  | agomir-NC: UUCUCCGAACGUGUCACGUTT |  |
|  | antagomir-NC: CAGUACUUUUGUGUAGUACAA |  |
| *DcVg1* (XM_008488883.3) | qF: CACCTACTCCTTGTCCTCTA | 166 bp |
|  | qR: GAAAAATCCCCAGAGTCCTT |  |
| *DcVg2* (XM_026832896.1) | qF: CTCCTCAGAAAGTGGAAGTT | 132 bp |
|  | qR: TTGTTTCCGATGAAGTAGGG |  |
| U6 | AGGATGACACGCAAAATCGT |  |
| *DcMet* (OP251123) | qF: AAGCTCAAGGGCCAAGTCAT | 257 bp |
|  | qR: TAATCAATACCAGGGGCGGC |  |
|  | RNAi-F: GATCACtaatacgactcactatagggCTTCATCTGTCGTATCAAGGT | 385 bp |
|  | RNAi-R: GATCACtaatacgactcactatagggATGAACTCTGCGTTGTTACT |  |
| *DcJHAMT* (KM212828.1) | qF: GTTCCTACTCCTATCGAACG | 190 bp |
|  | qR: ATCTGCGTGTAATTGAACCT |  |
|  | RNAi-F: GATCACtaatacgactcactatagggCCCTATACGAGACCAACAAC | 339 bp |
|  | RNAi-R: GATCACtaatacgactcactatagggGAACCCAGTGAAGACAGTAG |  |
| *DcECR* (XM_026829517.1) | qF: ACGAAGGCTCCTCGGAAGTCA | 194 bp |
|  | qR: AGTGGAGGTGGGTGTGGTGTAA |  |
|  | RNAi-F: GATCACtaatacgactcactatagggGTCAGGCGACGACCTATCTC | 522 bp |
|  | RNAi-R: GATCACtaatacgactcactatagggGCGAAGAGACCCGACTACTG |  |
| *DcVgR* (OP251122) | qF: AGCAGCTGGATATACATGTG | 186 bp |
|  | qR: CTCCACAGTACTGATTACCG |  |
| *Dicer-1* (XM_026830528.1) | qF: GTGGATGGATTAGAGTCTGC | 164 bp |
|  | qR: TTAGCCATATGTTGCGTGTA |  |
| *Ago-1* (XM_026830000.1) | qF: CTTTCCTAGTCGTGCAGAAA | 151 bp |
|  | qR: CTGACAAGGTAGAAGTCCAG |  |
| *DcNvd* (KAI5747348.1) | qF: TGAGTGTCCGTTTCATCAAT | 198 bp |
|  | qR: CTGGTGATCTTGTCCATAGG |  |
| *DcSpo* (XM_026821590.1) | qF: AAACACATGAACAAGATCGC | 170 bp |
|  | qR: ATGTGATCCCAAGACAACTC |  |
| *DcPhm* (KAI5718107.1) | qF: GGAAGATAGCCACTCATGAG | 227 bp |
|  | qR: GACATCTTGTTCCATAGCCA |  |
| *DcDib* (XM_017447442.2) | qF: TGGCTCACGGATTTATTGAA | 160 bp |
|  | qR: ATGTCCACACTCATACCAAC |  |
| *DcSad* (KAI5729656.1) | qF: AACAGCAATATCCACCCAAT | 217 bp |
|  | qR: CTTGGGAATCAGATAGCCTC |  |
| *DcShd* (XM_008489199.3) | qF: ATGAAGAGGACACTTGTTCC | 223 bp |
|  | qR: CCTGCCACATTGATGATTTC |  |

Note: The lowercase indicated the T7 promoter sequences in the primers of dsRNA synthesis. The sequences with black boxes displayed the restriction enzyme cutting sites. The underlines showed the homologous arm sequences for the seamless cloning in homologous recombination.
